# Taxonomy and pathogenicity of fungi associated with oak decline in northern and central Zagros forests of Iran with emphasis on coelomycetous species

**DOI:** 10.3389/fpls.2024.1377441

**Published:** 2024-04-18

**Authors:** Samaneh Bashiri, Jafar Abdollahzadeh

**Affiliations:** Department of Plant Protection, Faculty of Agriculture, University of Kurdistan, Sanandaj, Iran

**Keywords:** oak decline, pathogenicity, phylogeny, systematics, Zagros

## Abstract

Oak decline is a complex disorder that seriously threatens the survival of Zagros forests. In an extensive study on taxonomy and pathology of fungi associated with oak decline in the central and northern part of Zagros forests, 462 fungal isolates were obtained from oak trees showing canker, gummosis, dieback, defoliation, and partial or total death symptoms. Based on inter-simple sequence repeat (ISSR) fingerprinting patterns, morphological characteristics, and sequences of ribosomal DNA (28S rDNA and ITS) and protein coding loci (*acl1*, *act1*, *caM*, *tef-1α*, *rpb1*, *rpb2*, and *tub2*), 24 fungal species corresponding to 19 genera were characterized. Forty percent of the isolates were placed in eight coelomycetous species from seven genera, namely, *Alloeutypa*, *Botryosphaeria*, *Cytospora*, *Didymella*, *Gnomoniopsis*, *Kalmusia*, and *Neoscytalidium*. Of these, four species are new to science, which are introduced here as taxonomic novelties: *Alloeutypa iranensis* sp. nov., *Cytospora hedjaroudei* sp. nov., *Cytospora zagrosensis* sp. nov., and *Gnomoniopsis quercicola* sp. nov. According to pathogenicity trials on leaves and stems of 2-year-old Persian oak (*Quercus brantii*) seedlings, *Alternaria* spp. (*A. alternata*, *A. atra*, and *A. contlous*), *Chaetomium globosum*, and *Parachaetomium perlucidum* were recognized as nonpathogenic. All coelomycetous species were determined as pathogenic in both pathogenicity trials on leaves and seedling stems, of which *Gnomoniopsis quercicola* sp. nov., *Botryosphaeria dothidea*, and *Neoscytalidium dimidiatum* were recognized as the most virulent species followed by *Biscogniauxia rosacearum*.

## 1 Introduction

Oaks (*Quercus* L.) are the dominant vegetation of Zagros forests in western Iran with significant economic and environmental values (Heydari et al., 2013; Shiravand and Hosseini, 2020). Over the last two decades, oak decline as a serious ecological damage is spreading in this part of the world, which is caused by various biotic and abiotic factors affected by climate change, drought, wildfire, air pollution, irresponsible exploitation, and mismanagement (Ahmadi et al., 2014; Hosseini et al., 2018; Ahmadi et al., 2019; Shiravand and Hosseini, 2020; Mirhashemi et al., 2023). Fungi are well-known biotic agents, which affect trees under stress and cause canker, gummosis, vascular tissues necrosis, dieback, wilting, and partial and total death symptoms (Mirabolfathy et al., 2013; Ghasemi-Esfahlan et al., 2016, 2017; Safaee et al., 2017; Alidadi et al., 2019; Hanifeh et al., 2019; Moradi-Amirabad et al., 2019; Sabernasab et al., 2019; Di Lecce et al., 2020; Bashiri et al., 2020a, b, 2022). A wide variety of fungal species are associated with declined woody plants including coelomycetous fungi, a well-known morphological group belonging to the class *Dothideomycetes* (Wijayawardene et al., 2016). The most common pathogenic coelomycetous fungal species in association with declined trees showing canker, gummosis, dieback, wilting, and wood discoloration and necrosis are members of the *Botryosphaeriaceae* (e.g., *Botryosphaeria*, *Diplodia*, *Lasiodiplodia*, and *Neofusicoccum* spp.) (Alves et al., 2004; Turco et al., 2006; Linaldeddu et al., 2013; Dreaden et al., 2014; Smahi et al., 2017; Mahamedi et al., 2020), *Diatrypaceae* (e.g., *Diatrype*, *Diatrypella*, *Eutypa*, and *Eutypella* spp.) (Luque et al., 2000; Acero et al., 2004; Vasilyeva and Stephenson, 2004; Trouillas and Gubler, 2010; Lynch et al., 2013, 2014), *Cytosporaceae* (e.g., *Cytospora* spp.) (Preston, 1945; Spaulding, 1961; Senanayake et al., 2017; Shang et al., 2020, 2020; Pan et al., 2021), *Gnomoniaceae* (e.g., *Discula* and *Gnomoniopsis* spp.) (Moricca and Ragazzi, 2008; Sogonov et al., 2008; Walker et al., 2010; Jiang et al., 2021), and *Graphostromataceae* (e.g., *Biscogniauxia* spp.) (Raimondo et al., 2016; Safaee et al., 2017; Bahmani et al., 2021; Bashiri et al., 2022). Several studies on fungi associated with oak decline, mainly in the central part of Zagros forests, have introduced different fungal species most commonly belonging to the *Cytosporaceae*, *Diatrypaceae*, *Biscogniauxia*, and phoma-like genera (Mirabolfathy et al., 2013; Mehrabi et al., 2015; Ghobad-Nejhad et al., 2017, 2016, 2019; Alidadi et al., 2018, 2019; Ghasemi-Esfahlan et al., 2019; Sabernasab et al., 2019; Bashiri et al., 2022). In an extensive research on taxonomy and pathogenicity of fungal species associated with oak trees showing canker, gummosis, defoliation, wilting, dieback, and partial or total death symptoms in central and northern parts of Zagros forests, we identified 24 fungal species including 8 coelomycetous species. In this paper, we focus on the taxonomy and pathology of these species and introduce four new species as taxonomic novelties.

## 2 Materials and methods

### 2.1 Sampling, disease symptoms, and isolation of fungi

During a survey from July to November 2017, twigs and branches of oak trees showing external disease symptoms, dieback, wilting, canker, gummosis and twig/branch discoloration, and bark cracking were collected from Zagros forests of five provinces: Ilam, Kermanshah, Kurdistan, Lorestan, and West Azarbaijan. Cross-sections of the samples were examined to classify internal disease symptoms. To isolate fungi, small pieces of disinfested woody tissues (with 70% ethanol for 3 min) were transferred on potato dextrose agar (PDA) supplemented with 100 mg/L streptomycin sulfate and ampicillin and incubated at 20–25°C. Pure cultures were obtained using single spore or hyphal tip methods on tap water agar (2% WA). The isolates were stored on PDA at 4°C. Representative isolates were deposited in the culture collection of the Iranian Research Institute of Plant Protection (IRAN, Tehran, Iran) and the CBS collection of the Westerdijk Institute, Utrecht, The Netherlands. Isolates that were sequenced and studied morphologically are available in **Table 1**.

**Table 1:** Isolates, frequency, location, host, and collection codes of identified fungal species, disease symptoms observed and sequences generated in this study.

Species (no./frequency)	Location	Isolate no.^1^	Host	Disease symptoms^2^		GenBank accession number^3^					
				External	Internal	LSU	ITS	*tub2*	*tef-1α*	*act1*	*rpb2*
*A. iranensis* (18/3.9%)	Kermanshah, Kurdistan, Lorestan	IRAN 4323C^T^	*Q. brantii*, *Q. libani*	BC, BD	BH, CN, IrN	–	OR540613	OR591157	–	–	–
*B. dothidea* (19/4.1%)	Ilam, Kermanshah, Kurdistan, Lorestan, West Azarbaijan	IRAN 4314C CJASB115 CJASB108	*Q. brantii*, *Q.infectoria*, *Q. libani*	BD, BC, CB, DB, DF	BH, BS, BWS, CN, IrN, WN, W-SN	–	OR554270 OR554271 OR554272	- -	OR561900 OR561901 OR561902	- - -	- - -
*C. zagrosensis* (24/5.2%)	Kermanshah, Kurdistan, Lorestan	IRAN 4324C^T^ IRAN 4326C	*Q. brantii*, *Q.infectoria*, *Q. libani*	BD, BC, CB, DB, DF	Ar-SN, BS, W-SN, IrN	OR540615 OR540616	OR540618 OR540619	OR561905 OR561906	OR561993 OR561994	OR550646 OR550647	OR540303 OR540304
*C. hedjaroudei* (22/4.8%)	Ilam, Kermanshah, Kurdistan, Lorestan, West Azarbaijan	IRAN 4325C^T^	*Q. brantii*, *Q.infectoria*, *Q. libani*	BD, BC, DB, DF	Ar-SN, BH, IrN, WN	–	OR540617	OR561904	OR561995	–	–
*D. glomerata* (16/3.5%)	Kermanshah, Kurdistan, Lorestan	IRAN 4317C	*Q. brantii*, *Q. libani*	BC, BD	BH, BS, IrN,W-SN	–	–	OR558951	–	–	–
*G. quercicola* (28/6.1%)	Ilam, Kermanshah, Kurdistan, West Azarbaijan	IRAN 4313C^T^	*Q. brantii*, *Q.infectoria*, *Q. libani*	BC, BD, DB, DF, LC	BH, BS, CN, Ir-N, W-SN, WN	–	OR540614	OR561907	OR561996	–	–
*K. variispora* (37/8%)	Kermanshah, Kurdistan, Lorestan, West Azarbaijan	IRAN 4318C IRAN 4710C	*Q. brantii*, *Q.infectoria*, *Q. libani*	BC, BD, DF	BH, BS, Ir-N	- -	OR551504 OR551505	- -	- -	- -	- -
*N. dimidiatum* (20/4.3%)	Ilam, Kermanshah, Lorestan	IRAN 4312C	*Q. brantii*	BD, BC, CB, DB	BH, BS, CN, Ir-N	–	OR554390	–	OR561903	–	–

^1^ Sequenced isolates, IRAN, Iranian Fungal Culture Collection, Iranian Research Institute of Plant Protection, Iran; T, Ex-type. ^2^ Disease symptoms observed on oak trees in the forest, BC, Branch Canker; BD, Branch Dieback; CB, Cracked Bark; DB, Discoloration of Bark; LC, Leaf Chlorosis; DF, Defoliation; Ar-SN, Arch-shaped Necrosis; BH, Borer Hole; BS, Black Spots; CN, Central Necrosis; BWS, Black or Brown Wood Streaks; IrN, Irregular Necrosis; WN, Watery Necrosis; W-SN, Wedge-Shaped Necrosis. ^3^ LSU, partial 28S large subunit RNA gene; ITS, internal transcribed spacers and intervening 5.8S nrDNA gene (ITS) of the nrDNA operon; tub2, partial β-tubulin gene; tef-1α, partial translation elongation factor 1-alpha gene; rpb2, partial RNA polymerase II second largest subunit gene; act1, partial actin gene; - indicates not sequenced.

### 2.2 Molecular experiments

Total DNA was extracted from isolates grown on potato dextrose broth (PDB) after 7–10 d at 25°C using the modified Raeder and Broda (1985) method as described by Abdollahzadeh et al. (2009). To reduce the number of isolates for sequencing and microscopy, fungal isolates were grouped at the species level using the inter-simple sequence repeat (ISSR) technique based on DNA profiles generated with M13 primer (data not shown), and representative isolates of each group were sequenced. According to fungal taxonomic groups or genera deduced from morphology, different gene regions were amplified and sequenced using the following primer pairs: LROR/LR5 for a part of 28S nrDNA (LSU) (Vilgalys and Hester, 1990; Cubeta et al., 1991), ITS5/ITS4 for the ITS1-5.8S nrDNA-ITS2 region (ITS) (White et al., 1990), ACT512F/ACT783R for a part of actin (*act1*) (Carbone and Kohn, 1999), 5F (or 5F2)/7cR for a part of RNA polymerase II second largest subunit (*rpb2*) (Woudenberg et al., 2013), EF1-728F/EF1-986R (Vilgalys and Hester, 1990) for a part of translation elongation factor 1-alpha (*tef-1α*), and Bt2a (or T1)/Bt2b for a part of β-tubulin (*tub2*) (O’Donnell and Cigelnik, 1997; Glass and Donaldson, 1999). The PCR mixtures (25 µL) consisted of 1×PCR buffer, 3 mM MgCl_2_, 200 µM of dNTPs, 5 pmol of each primer, 1 U of *Taq* DNA polymerase, and 1 µL of template DNA (50–100 ng/µL). For amplification of ITS and *tub*2 regions, we followed the PCR conditions as described by Bashiri et al. (2022). The PCR conditions for *tef-1α* and *rpb2* were as follows: an initial denaturation step of 5 min at 95°C followed by 35 cycles of 30 s at 95°C, 30 s at 52°C (*tef-1α*)/45 s at 54°C (*rpb2*)/1 min at 55°C (LSU)/30 s at 58°C (*act1*), and 90 s at 72°C, with a final extension of 7 min at 72°C. PCR products were purified and sequenced by Elim Biopharm (USA) via FAZABiotec Co. (Tehran, Iran) and BGI (China) via BMG (Bio Magic Gene) Co. (Karaj, Iran). Consensus sequences were prepared with BioEdit v. 7.0.0 (Hall, 2004). All new sequences generated in this study were submitted to GenBank (**Table 1**).

### 2.3 Phylogeny

The generated sequences together with the sequences retrieved from GenBank were aligned using Clustal X v. 1.83 (Thompson et al., 1997) or online MAFFT v. 7 and edited manually in BioEdit v. 7.0.0, where necessary. Each locus was aligned separately and the alignments were concatenated with Mesquite 2.75 (Maddison and Maddison, 2023). Both single and combined loci were analyzed by Maximum Parsimony (MP), Maximum Likelihood (ML), and Bayesian Inference (BI). MP was performed using PAUP v. 4.0b10 (Swofford, 2003). ML and BI were carried out through the online CIPRES Science Gateway (Miller et al., 2012) using RAxML-HPC BlackBox v. 8.2.10 (Stamatakis, 2014) and MrBayes v. 3.2.6 (Huelsenbeck and Ronquist, 2001; Ronquist and Huelsenbeck, 2003), respectively. MP analysis was executed according to Abdollahzadeh et al. (2013). Optimal nucleotide substitution models were detected for each locus using MrModelTest v. 2.3 (Nylander, 2004). The ML and Bayesian analyses were executed as described by Abdollahzadeh et al. (2020). Phylograms were plotted using FigTree v. 1.4.3 and edited in Adobe Illustrator CS2 v. 12.0.0. Alignments and trees were deposited in TreeBASE (www.treebase.org; S30794, S30795, and S30796) and taxonomic novelties registered in MycoBank (Crous et al., 2004).

### 2.4 Phenotypic and microscopic studies

Depending on the fungal morphological group, culture characteristics and microscopic fungal structures were examined and recorded from cultures grown on PDA, oat meal agar (OA), malt extract agar (MEA), and yeast malt agar (YMA) at room temperature. Sporulation and fruiting body production were induced under a combination of near-UV and cool-white fluorescent lights under 12-h light/12-h dark conditions. The structure and dimensions of microscopic features were determined and measured in 100% lactic acid or distilled water using an Olympus DP72 camera on an Olympus BX51 microscope and a Cell Sense Entry measurement module. To compute the dimensions of each fungal structure, mean, standard deviation, and 95% confidence intervals were estimated based on at least 30 microscopic measurements. Dimensions are presented as the range of measurements with extremes in brackets followed by mean ± standard deviation.

### 2.5 Pathogenicity tests

Pathogenicity tests of recognized species were performed on leaves and stems of 2-year-old oak (*Q. brantii*) seedlings in Petri plates and under greenhouse conditions, respectively. The surface of leaves and stems was disinfested with 70% ethanol. Small pieces of bark (≈ 3 × 3 mm) were cut from the stems of potted seedlings and small scratches (≈3 mm) were made on the leaves. Mycelial plugs of 7-d-old colonies on PDA were inoculated on wounded leaves and stems. Controls were inoculated with sterile PDA plugs. To evaluate the phytotoxic activity of fungal isolates, inoculated leaves were incubated at 25°C for a period of 7 days. The inoculated seedling wounds were wrapped with Parafilm and placed under greenhouse conditions (22–28°C) and watered as needed. As the seedlings declined, the external symptoms were recorded and the extent of vascular discoloration (lesion length) was measured. To confirm Koch’s postulates, fungal isolates were re-isolated from inoculated leaves and stems on PDA at 25°C and morphologically re-examined. The greenhouse trials were performed as a completely randomized nested design (CRND) with three replications per treatment. To determine differences in lesion lengths caused by inoculated fungi, one-way ANOVA was executed. Homogeneity of variance and normality assumption were examined using Bartlett and Shapiro–Wilk’s tests, respectively. Least significant difference (LSD) values were calculated (*p* = 0.05) using SAS v. 9.1.3.

## 3 Results

### 3.1 Disease symptoms, fungal isolates, and species identification

**Figure 1:**
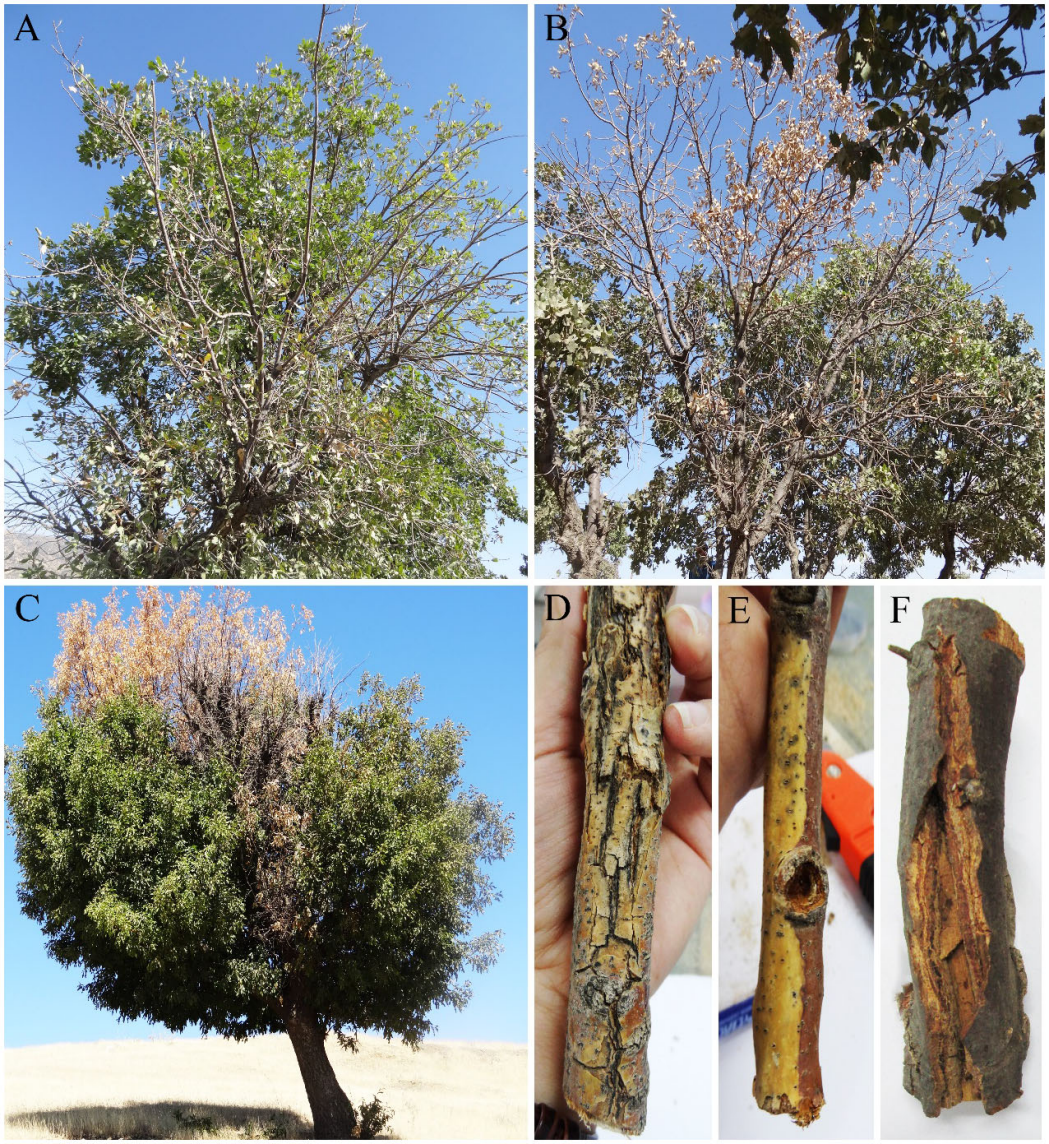
External symptoms on oak trees. **(A)** Defoliation, **(B, C)** dieback, and **(D–F)** cankers and discoloration on twigs and branches.

**Figure 2:**
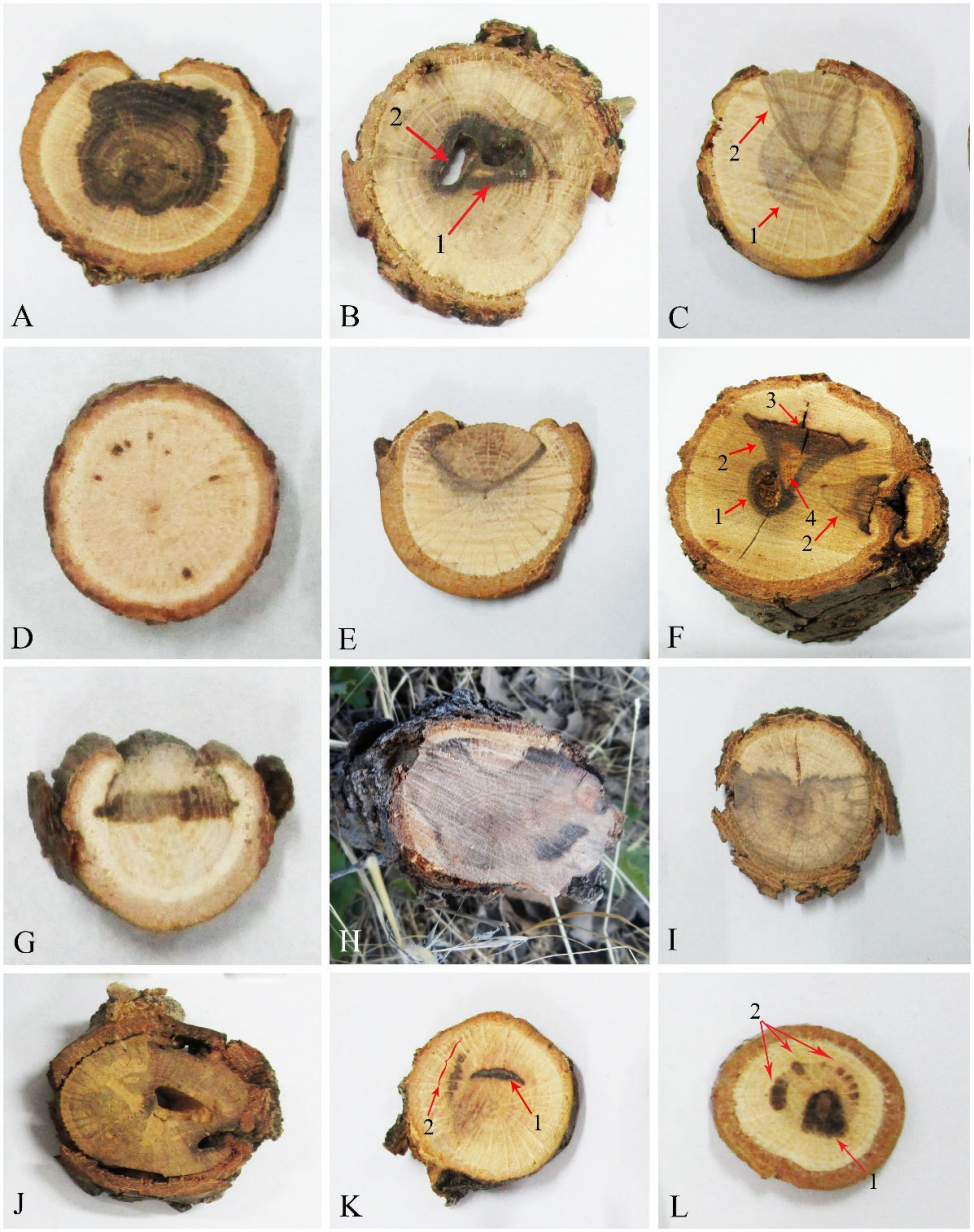
Internal wood symptoms in cross-sections of trunks and branches of oak trees. **(A, H, I)** Extended irregular necrosis. **(B)** Co-occurrence of central necrosis (1) and borer holes (2). **(C)** Co-occurrence of central necrosis (1) and wedge-shaped necrosis. **(D)** Black spots. **(E)** Wedge-shaped necrosis. **(F)** Co-occurrence of borer hole (1), wedge-shaped necrosis (2), black wood streaking (3), and wood decay (4). **(G)** Irregular necrosis. **(J)** Discolored wood areas surrounding borer holes. **(K)** Co-occurrence of arch-shaped necrosis (1) and black spots (2). **(L)** Co-occurrence of central necrosis (1) and black spots (2).

A total of 462 fungal isolates were collected from oak trees (*Q. brantii*, *Q. infectoria*, and *Q. libani*), of which 184 isolates (40%) were accommodated in coelomycetous *Dothideomycetes*. Various external and internal symptoms (**Figures 1**, **2**) were observed on trees’ and twigs’ cross-sections listed in **Table 1**. Primarily, borer feeding sites (e.g., *Buprestidae*) were observed in association with irregular and central wood necrosis and no correlation was found between fungal species and type of disease symptoms.

According to DNA profiles generated with M13 primer (data not shown), 36 isolates as representatives of recognized DNA banding patterns were selected for morphological and phylogenetic analyses.

**Figure 3:**
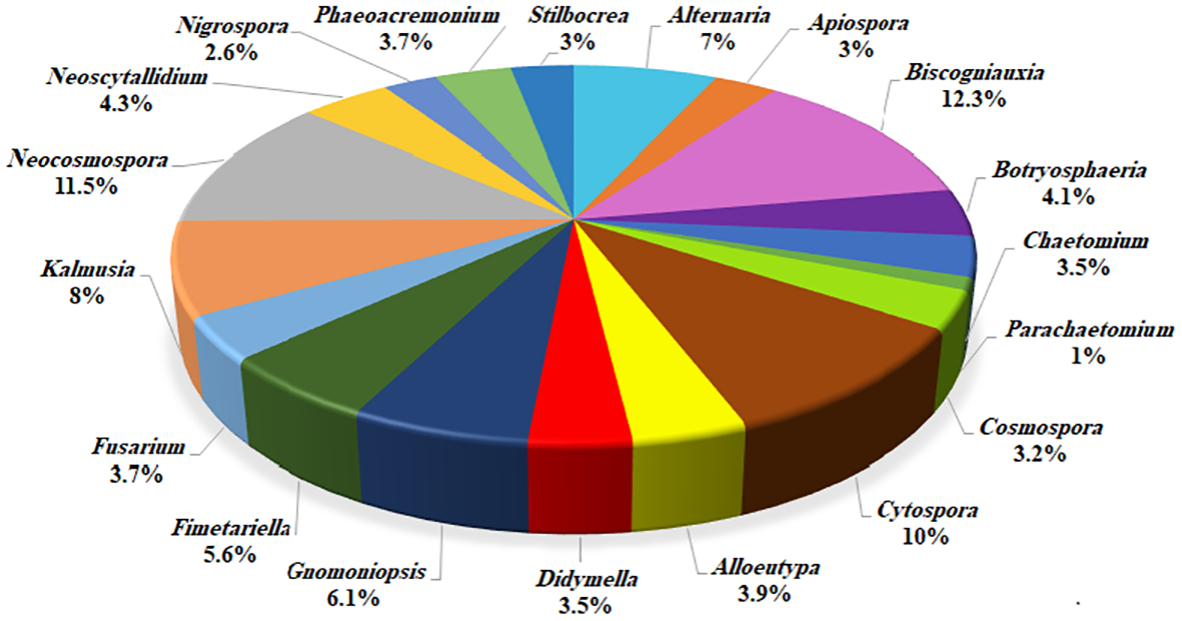
Distribution and frequency of identified fungi at the generic level.

**Figure 4:**
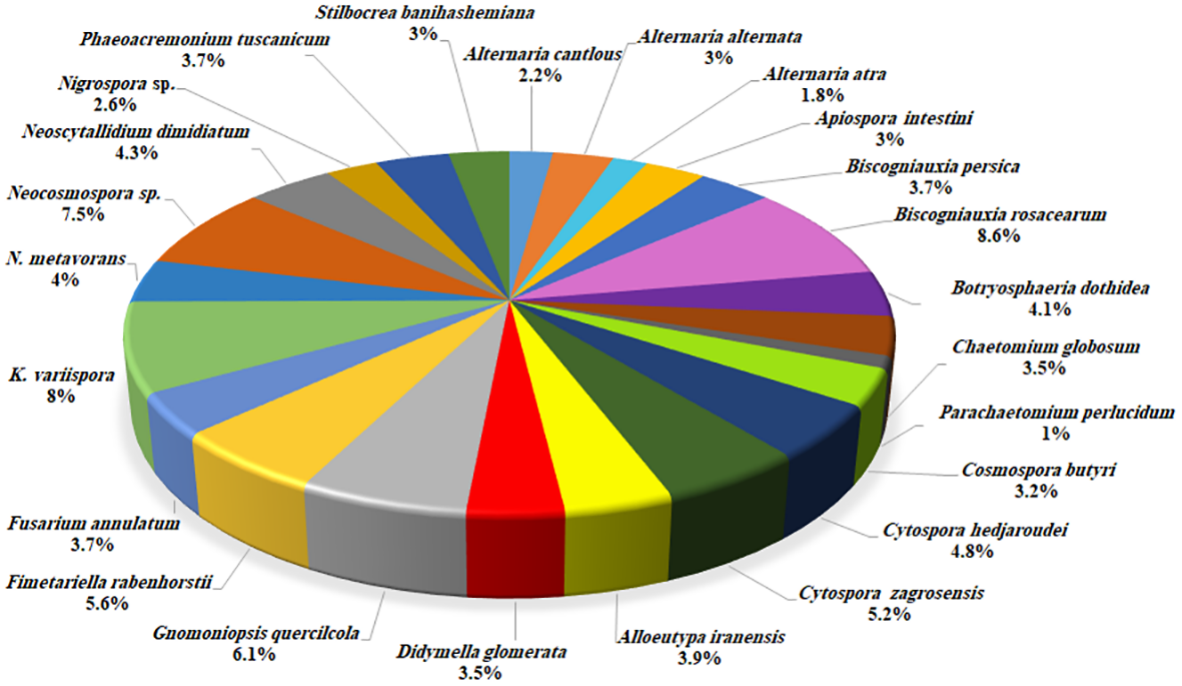
Distribution and frequency of identified fungal species.

Based on morphology and DNA sequence data (LSU, ITS, *rpb1*, *rpb2*, *tef-1α*, *tub2*, *acl1*, *act1*, and *caM*), 24 fungal species corresponding to 19 genera were recognized (**Figures 3**, **4**). Of these, eight species with 184 isolates (40%) were placed in coelomycetous *Dothideomycetes*, namely, four known species—*Botryosphaeria dothidea* (19 isolates/4.1%), *Didymella glomerata* (16 isolates/3.5%), *Kalmusia variispora* (37 isolates/8.1%), and *Neoscytalidium dimidiatum* (20 isolates/4.3%)—and four new species that are described and named *Alloeutypa iranensis* sp. nov. (18 isolates/3.9%), *Cytospora hedjaroudei* sp. nov. (22 isolates/4.8%), *Cytospora zagrosensis* sp. nov. (24 isolates/5.2%), and *Gnomoniopsis quercicola* sp. nov. (28 isolates/6.1%) (**Table 1**, **Figure 4**). *Biscogniauxia*, *Neocosmospora*, and *Cytospora* were the most prevalent fungi with frequencies of 12.3%, 11.5%, and 10%, respectively (**Figure 3**). Among the coelomycetous fungi, *Cytospora* and *Kalmusia* were the most common fungi associated with the decline of oak trees followed by *Gnomoniopsis*, *Neoscytalidium*, *Botryosphaeria*, *Alloeutypa*, and *Didymella* (**Figure 3**). Moreover, *Biscogniauxia rosacearum*, *K. variispora*, and *Neocosmospora* sp. were the most prevalent fungi at the species level followed by *G. quercicola*, *Fimetariella rabenhorstii*, *C. zagrosensis*, and *C. hedjaroudei* (**Figure 4**).

### 3.2 Phylogenetic analyses

Megablast search of the GenBank nucleotide database with ITS sequences revealed that our isolates are close to the members of *Diatrypaceae* (*Eutypa*/*Eutypella*), *Cytosporaceae* (*Cytospora*), and *Gnomoniaceae* (*Discula*/*Gnomoniopsis*). Thus, based on blast searches, preliminary phylogenetic analyses, literature, and fungal databases (indexfun-gorum/speciesfungorum/mycobank), three datasets were provided and analyzed.

**Figure 5:**
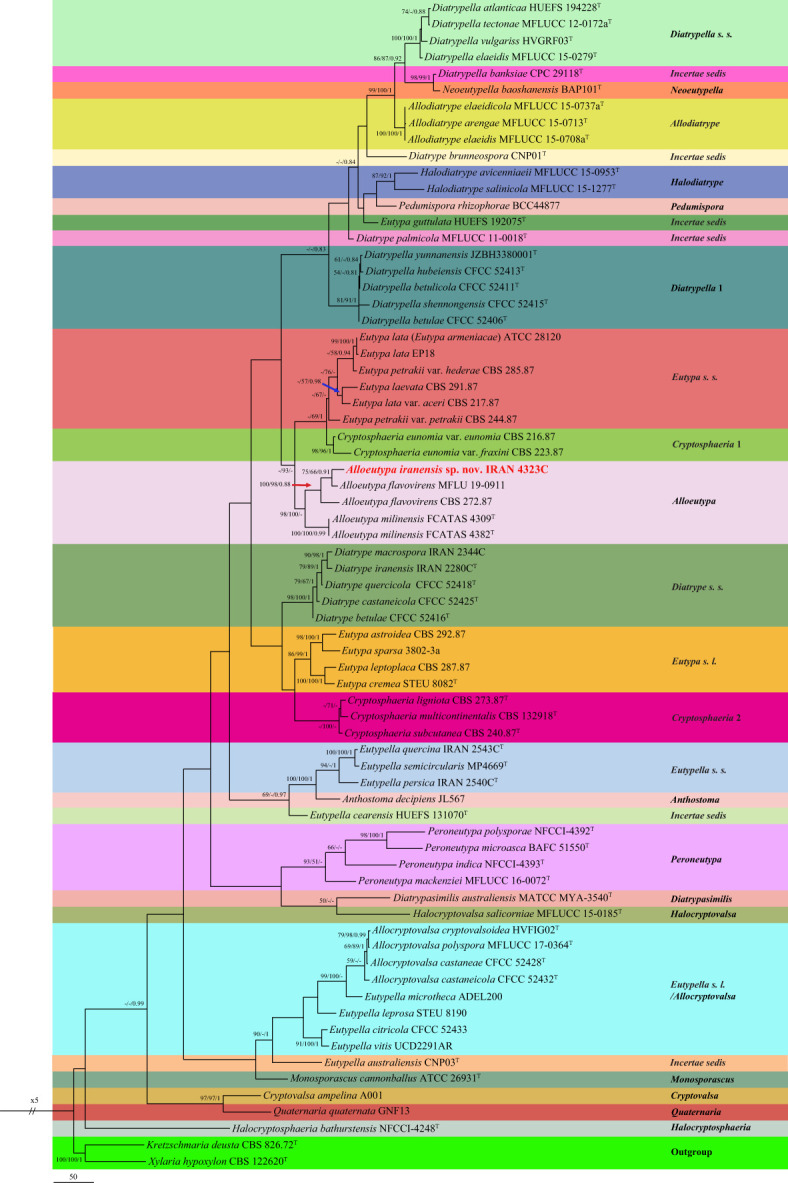
One of the 729 most parsimonious trees of *Diatrypaceae* family obtained from analyses of combined ITS and *tub2* sequence data. MP/ML/BI bootstrap support values and posterior probabilities are shown at the nodes. The phylogenetic tree was rooted with *Kretzschmaria deusta* CBS 826.72/*Xylaria hypoxylon* CBS 122620. The novel taxon is in boldface. ^T^ Ex-type.

The first DNA sequence dataset (ITS: 71, *tub2*: 47 sequences) consisted of our selected isolate IRAN 4323C, 68 isolates belonging to 66 species of the *Diatrypaceae* family, and two outgroups *Xylaria hypoxylon* CBS 122620/*Kretzschmaria deusta* CBS 826.72. The aligned datasets of ITS (691) and *tub2* (454) were combined and subjected to MP, ML, and BI. After alignment, the combined dataset consisted of 1,145 characters including alignment gaps. Of these, 490 were constant, 192 were variable and parsimony-uninformative, and 463 were parsimony-informative. MP analysis of the remaining 463 parsimony-informative characters resulted in 729 most parsimonious trees (TL = 2,925, CI = 0.41, RI = 0.6, HI = 0.59). MrModelTest revealed that the general time-reversible model of evolution (Rodríguez et al., 1990), including estimation of invariable sites and assuming a discrete gamma distribution (GTR+I+G) with six rate categories (lsetnst = 6, rates = invgamma) and dirichlet (1,1,1,1) base frequencies, is the best nucleotide substitution model for both loci (ITS, *tub2*). The Bayesian analyses of the concatenated alignments of two loci generated 3,682 trees from which 920 trees were discarded as burn-in. The consensus tree and posterior probability values (PP) were calculated from the remaining 2,762 trees. The average standard deviation of split frequencies was 0.009962 at the end of the run. The RAxML search of the dataset with 760 distinct alignment patterns produced a best-scoring ML tree (lnL = −14,040.126555). The ML and Bayesian phylogenetic trees were mapped on the MP tree shown in **Figure 5** with MP/ML/BI bootstrap support and posterior probability values at the nodes. In these analyses, our isolate IRAN 4323C was placed in a distinct clade in *Alloeutypa*, a new genus recently introduced in *Diatrypaceae*, which is recognized here as a new species named *Alloeutypa iranensis* sp. nov. close to *A. flavovirens* CBS 272.87 and isolate MFLU 19-0911 (**Figure 5**). *A. iranensis* showed remarkable differences in nucleotide sequences with *A. flavovirens* CBS 272.87 (ITS: 7 substitutions; *tub2*: 7 substitutions, 21 deletions/insertions) and MFLU 19-0911 (ITS: 5 substitutions; *tub2*: 6 substitutions, 25 deletions/insertions). It is also significantly distinct from the type species *A. milinensis* FSATAS 4309 based on ITS (12 substitutions, 1 deletion/insertion) and *tub2* (16 substitutions, 25 deletions/insertions) sequence data. Isolate MFLU 19-0911 is placed in a distinct clade and is apparently a representative of a new *Alloeutypa* species.

**Figure 6:**
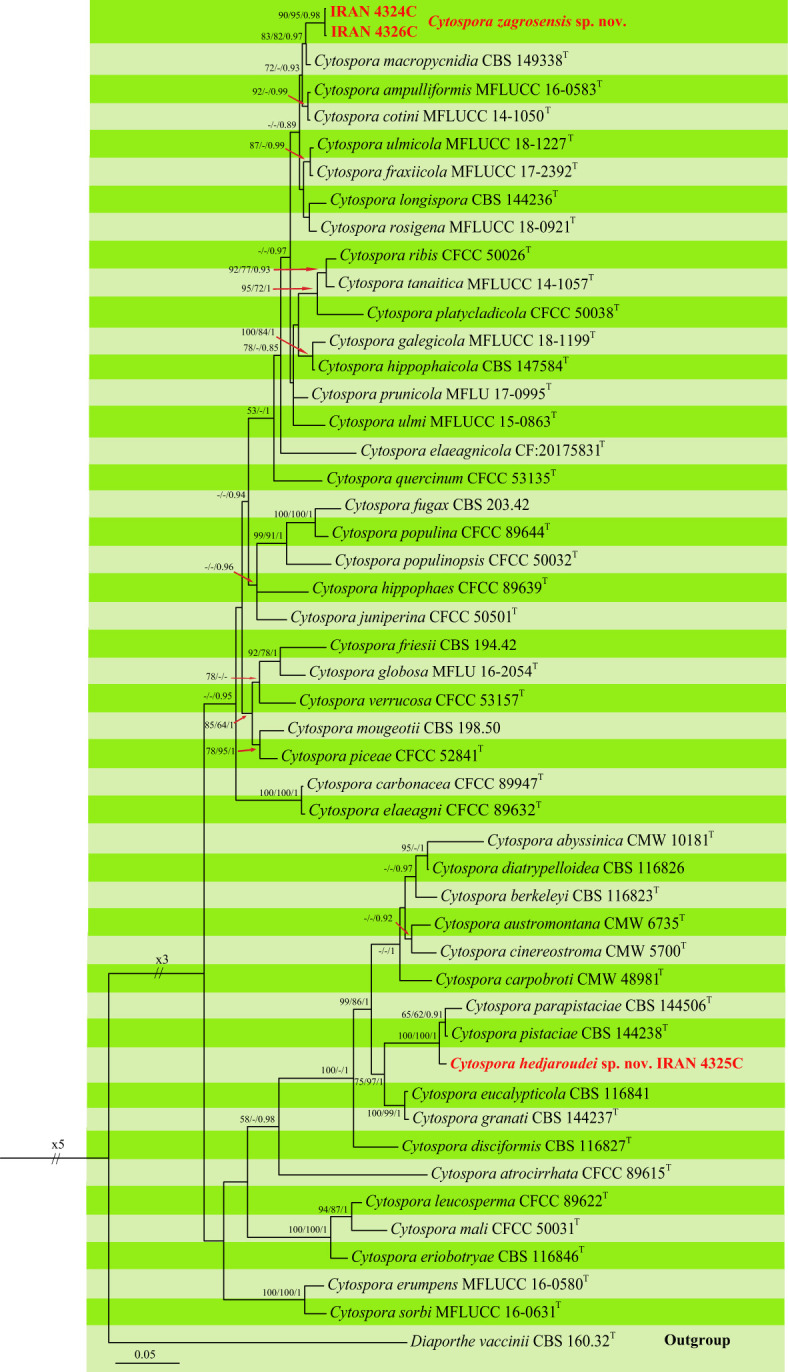
Phylogenetic tree of *Cytospora* spp. inferred from Bayesian analysis of combined LSU, ITS, *rpb2*, *ef1-a*, *act1*, and *tub2* sequence data. MP/ML/BI bootstrap support values and posterior probabilities are shown at the nodes. The phylogenetic tree was rooted with *Diaporthe vaccinii* CBS 160.32. The new species are in boldface. ^T^ Ex-type.

The second dataset consisting of DNA sequences of six loci (LSU, ITS, *rpb2*, *act1*, *tef-1α*, and *tub2*) was analyzed to examine the taxonomic position of our *Cytospora* isolates. New generated sequences were aligned with available authentic sequences of *Cytospora* species and *Diaporthe vaccinii* CBS 160.32 as an outgroup (LSU: 43, ITS: 47, *rpb2*: 41, *act1*: 44, *tef-1α*: 39, *tub2*: 29 sequences). The concatenated alignment of LSU (803), ITS (593), *rpb2* (664), *act1* (311), *tef-1α* (420), and *tub2* (503) was subjected to MP, ML, and BI. After alignment, the dataset consisted of 3,295 characters including alignment gaps. Of these, 2,283 were constant, 222 were variable and parsimony-uninformative, and 790 were parsimony-informative. MP analysis of the remaining 790 parsimony-informative characters resulted in 162 most parsimonious trees (TL = 3,345, CI = 0.48, RI = 0.67, HI = 0.52). MrModelTest revealed that the general time-reversible model of evolution (Rodríguez et al., 1990), including estimation of invariable sites and assuming a discrete gamma distribution (GTR+I+G) with six rate categories (lsetnst = 6, rates = invgamma) and dirichlet (1,1,1,1) base frequencies, is the best nucleotide substitution model for all loci (LSU, ITS, *rpb2*, *act1*, *tef-1α*, and *tub2*). The Bayesian analyses of the concatenated alignments of six loci generated 1,172 trees from which 292 trees were discarded as burn-in. The consensus tree and posterior probability values (PP) were calculated from the remaining 880 trees. The average standard deviation of split frequencies was 0.009890 at the end of the run. The RAxML search of the dataset with 1,272 distinct alignment patterns produced a best-scoring ML tree (lnL = −20,279.905622). The MP and ML trees were mapped on the Bayesian phylogenetic tree as shown in **Figure 6** with MP/ML/BI bootstrap support and posterior probability values at the nodes.

Our isolates were placed in two distinct clades differentiated from all other *Cytospora* species that are recognized here as two new species. Two isolates, IRAN 4324C and IRAN 4326C, formed a well-supported clade as a sister group with *C. macropycnidia* CBS 149338 and named *Cytospora zagrosensis* sp. nov. This species is differentiated from *C. macropycnidia* in ITS (2 substitutions), *rpb2* (2 substitutions), *tef-1α* (6 substitutions, 2 deletions/insertions), and *tub2* (11 substitutions, 3 deletions/insertions) nucleotide sequences. The third isolate IRAN 4325C represents the second new species close to *C. pistaciae* CBS 144238 and *C. parapistaciae* CBS 144506, which is named *Cytospora hedjaroudei* sp. nov. (**Figure 6**). Based on nucleotide sequences, *C. hedjaroudei* differs from two closely related species *C. pistaciae* (ITS: 5 substitutions, 1 deletion/insertion; *tef-1α* 1 substitution) and *C. parapistaciae* (ITS: 8 substitutions, 28 deletions/insertions; *tef-1α*: 4 substitutions, 1 deletion/insertion). It is noticeable that ITS sequence data are enough to separate *C. hedjaroudei* from both *C. pistaciae* and *C. parapistaciae*.

**Figure 7:**
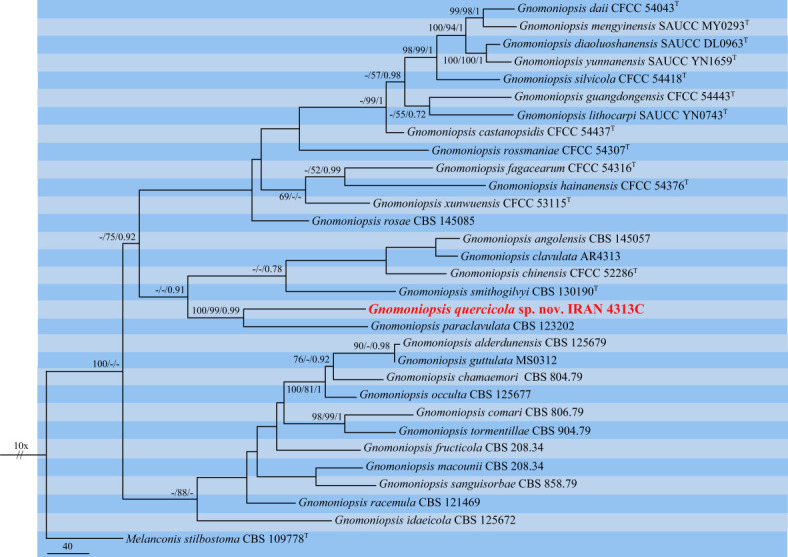
One of the three most parsimonious trees of *Gnomoniopsis* spp. obtained from combined ITS, *tef-1α*, and *tub2* sequence data. MP/ML/BI bootstrap support values and posterior probabilities are shown at the nodes. The phylogenetic tree was rooted with *Melanconis stilbostoma* CBS 109778. The novel species is in boldface. ^T^ Ex-type.

The third dataset contained three loci DNA sequences (ITS: 31, *tef-1α*: 23, and *tub2*: 26 sequences) of our selected isolate IRAN 4313C, 29 *Gnomoniopsis* species, and *Melanconis stilbostoma* CBS 109778 as an outgroup. The aligned single gene sequences were concatenated and subjected to MP, ML, and BI. The combined dataset consisted of 1,918 characters (ITS: 606, *tef-1α*: 370, and *tub2*: 942) including alignment gaps. Of these, 1,099 were constant, 278 were variable and parsimony-uninformative, and 541 were parsimony-informative. MP analysis of the remaining 541 parsimony-informative characters resulted in three most parsimonious trees (TL = 2,367, CI = 0.55, RI = 0.6, HI = 0.45). MrModelTest revealed that the general time-reversible model of evolution (Rodríguez et al., 1990), including estimation of invariable sites and assuming a discrete gamma distribution (GTR+I+G) with six rate categories (lsetnst = 6, rates = invgamma) and dirichlet (1,1,1,1) base frequencies, is the best nucleotide substitution model for all loci (ITS, *tef-1α*, and *tub2*). The Bayesian analyses of the concatenated alignments of three loci generated 4,892 trees from which 1,222 trees were discarded as burn-in. The consensus tree and posterior probability values (PP) were calculated from the remaining 3,670 trees. The average standard deviation of split frequencies was 0.009837 at the end of the run. The RAxML search of the dataset with 976 distinct alignment patterns produced a best-scoring ML tree (lnL = −13,465.491131). The ML and Bayesian phylogenetic trees were mapped on the MP tree shown in **Figure 7**. Our isolate IRAN 4313C was clustered in a strongly supported clade close to *G. paraclavulata* CBS 123202 with significant nucleotide differences in all three loci ITS (5 substitutions, 2 deletions/insertions), *tef-1α* (35 substitutions, 8 deletions/insertions), and *tub2* (58 substitutions, 9 deletions/insertions). Thus, it is recognized as a new species and named *Gnomoniopsis quercicola* sp. nov. (**Figure 7**).

### 3.3 Taxonomy

**Figure 8:**
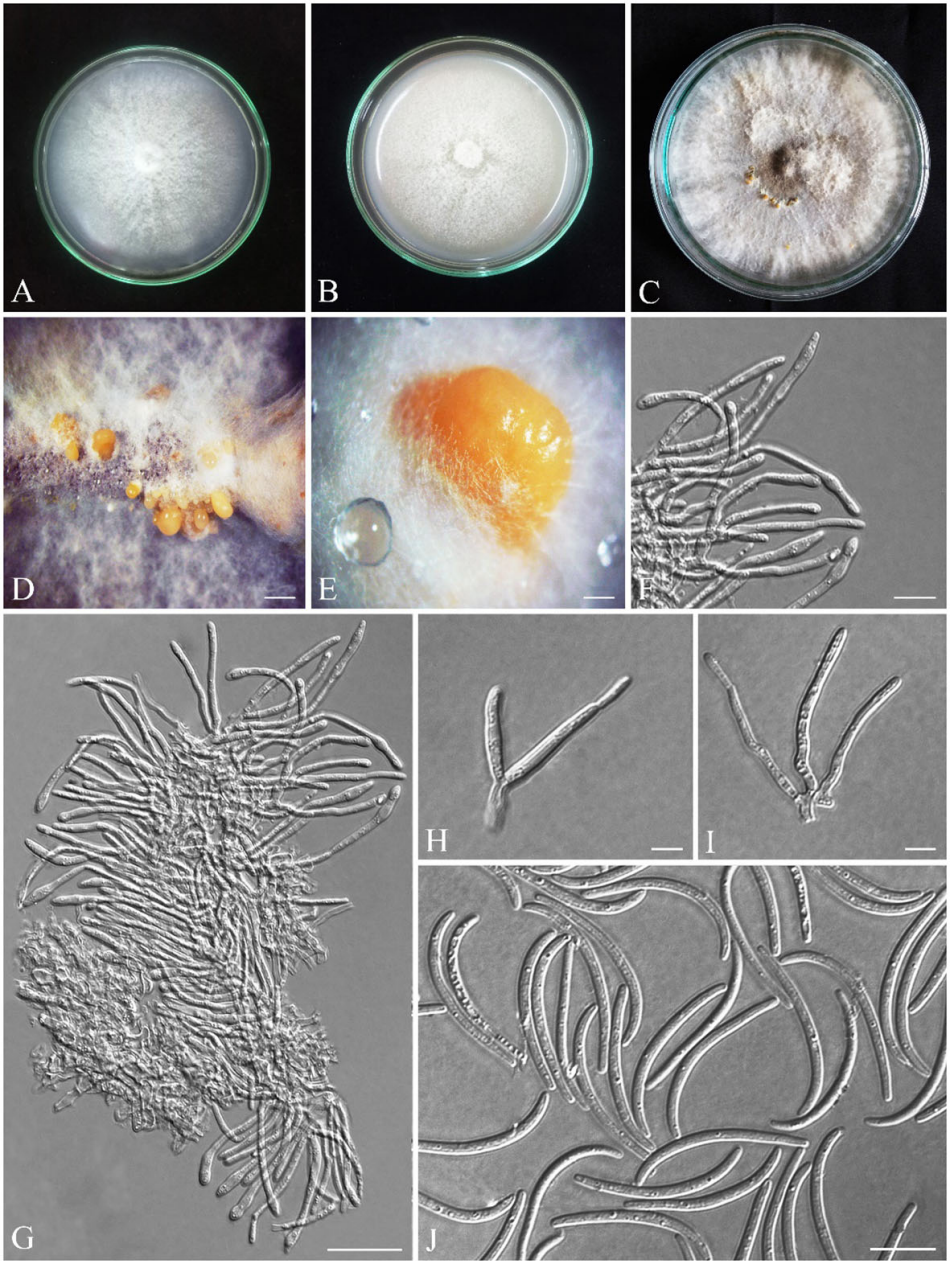
Seven-day-old colonies of *Alloeutypa iranensis* (IRAN 18255F, Holotype). **(A)** On PDA. **(B)** On OA. **(C)** Three-month-old colonies on MEA at room temperature. **(D, E)** Conidiomata. **(F–I)** Conidiophores and conidiogenous cells. **(J)** Conidia. Scale bars: **(D)** 900 μm; **(E)** 400 μm; **(F, H, I)** 5 μm; **(G, J)** 10 μm.

Based on phylogenetic analyses and morphology, eight coelomycetous fungal species were characterized and associated with declined oak trees in the central and northern part of Zagros forests in Iran. Of these, four species were recognized as new fungal species for science, which are described here as follows:

*Alloeutypa iranensis* S. Bashiri & Abdollahz., sp. nov. (**Figure 8**)

MycoBank: MB850401.

*Diagnosis*: *A. iranensis* is the third species to be described in *Alloeutypa*. Since the type species *A. milinensis* was described based on the sexual morph and we have only observed the asexual morph, it is only possible to morphologically distinguish *A. iranensis* from *A. flavovirens* based on the larger dimensions of conidiomata (0.9–3.9 mm vs. 0.7–1.3 mm), conidiophores (12.6 × 3 μm vs. 4.9 × 2.4 μm), and conidiogenous cells (32.4 × 2.6 μm vs. 11.5× 2.4 μm) and shorter conidia (28.5 μm vs. 34 μm).

#### 3.3.1 Etymology

*iranensis* refers to the country where the fungus was first found.

#### 3.3.2 Type

IRAN, Lorestan Province, Khorramabad, from *Q. brantii* branches, 33°18′50.2″ N/48°43′12.0″ E, 27 September 2016, S. Bashiri, IRAN 18255F (Holotype) (GenBank ITS: OR540613; *tub2*: OR591157), ex-type IRAN 4323C = CBS 149771.

#### 3.3.3 Description

#### 3.3.4 Sexual morph

not observed.

#### 3.3.5 Asexual morph

Pycnidia produced on MEA, superficial, solitary or aggregated, sub-conical, globose to sub-globose, shiny, with smooth surface, cream to pale yellow (**Figures 8D, E**). Peridium thick, comprising dark cream, thick-walled, cells of textura angularis. Conidiophores branched, arising from pseudoparenchymatous cells or interwoven hyphae, (5.6–) 11–16 (–17.1) × (2–) 2.4–4 (–5.4) μm (av. ± SD = 12.6 ± 0.5 × 3 ± 0.1 µm). Conidiogenous cells cylindrical, in dense palisades, straight or curved, apex mostly inflated, (18–) 24–43 (–51.7) × (1.8–) 2–3.5 (–3.9) μm (av. ± SD = 32.4 ± 1.3 × 2.6 ± 0.1 µm). Conidia filiform, curved or rarely straight, with blunt apex and flattened base, hyaline, (23.3–) 25–30 (–33.3) × (1.6–) 1.7–2 (–2.5) μm (av. ± SD = 28.5 ± 0.3 × 2 ± 0.02 µm) (**Figures 8F–J**).

#### 3.3.6 Culture characteristics

Colonies on PDA cottony, dense, white, margin smooth, reverse yellowish-white, reaching 75 mm diameter after 7 days at room temperature in the dark; on MEA flat, white and becoming grayish white with age, margin smooth, reverse grayish, reaching 60 mm diameter after 7 days at room temperature in the dark; on OA cottony, white and becoming grayish white with age, margin smooth, reverse grayish, reaching 90 mm diameter after 7 days at room temperature in the dark (**Figures 8A–C**). Mycelial clumps produced on MEA, erect, white, and floccose on culture.

#### 3.3.7 Additional specimens examined

Iran, Kurdistan Province, Baneh, from *Q. libani* branches, 22 August 2016, S. Bashiri, IRAN 4837C = CBS 149772 (36°05′01.8″ N/45°40′32.8″ E), CJASB222/CJASB223 (36°03′46.7″ N/45°38′32.3″ E); Iran, Kurdistan Province, Sarvabad, from *Q. libani* branches, 15 August 2016, S. Bashiri, CJASB225 (35°22′95.7″ N/46°12′89.5″ E), CJASB226 (35°09′87.1″ N/46°32′42.4″ E); Iran, Kurdistan Province, Marivan, from *Q. libani* branches, 15 August 2016, S. Bashiri, IRAN 4895C (35°24′78.4″ N/46°10′73.4″ E), CJASB228 (35°28′83.4″ N/46°06′22.8″ E); Iran, Kermanshah Province, Eslamabad-e-Gharb, from *Q. brantii* branches 11 October 2016, S. Bashiri, IRAN 4897 (33°58′68.9″ N/46°29′92.0″ E); Iran, Kermanshah Prov-ince, Paveh, from *Q. brantii* branches, 21 October 2016, S. Bashiri, CJASB230/CJASB231(34°57′06.9″ N/46°27′80.5″ E); Iran, Lorestan Province, Chegini, from *Q. brantii* branches, 27 September 2016, S. Bashiri, CJASB232 (35°32′49.7″ N/48°05′23.8″ E), IRAN 4896C/CJASB235 (35°33′76.3″ N/46°06′18.8″ E); Iran, Lorestan Province, Khorramabad, from *Q. brantii* branches, 25 September 2016, S. Bashiri, CJASB234 (33°39′40.4″ N/46°16′85.5″ E); Iran, Lorestan Province, Kuhdasht, from *Q. brantii* branches, 27 Sep-tember 2016, S. Bashiri, CJASB236 (33°31′28.4″ N/47°47′24.6″ E); Iran, Lorestan Province, Poldokhtar, from *Q. brantii* branches, 27 September 2016, S. Bashiri, CJASB237/CJASB238 (33°18′12.2″ N/47°48′95.0″ E).

**Figure 9:**
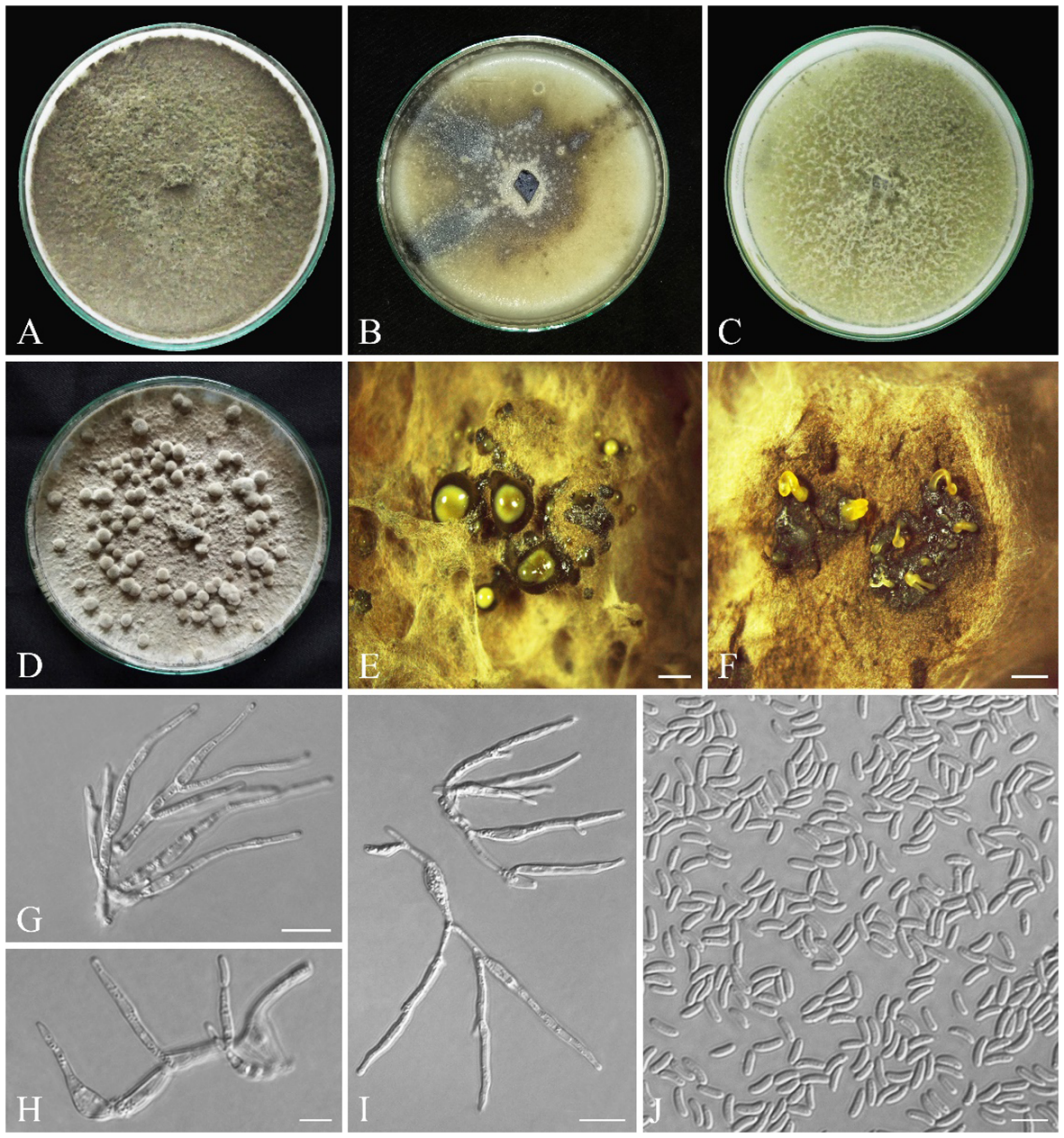
Seven-day-old colonies of *Cytospora hedjaroudei* (IRAN 18257F, Holotype). **(A)** On PDA. **(B)** On OA. **(C)** On MEA. **(D)** Fourteen-day-old colonies on PDA at room temperature. **(E)** Conidiomata. **(F)** Cross-section of conidiomata. **(G–I)** Conidiogenous cells. **(J)** Conidia. Scale bars: **(E, F)** 900 μm; **(G, I)** 10 μm; **(H, J)** 5 μm.

*Cytospora hedjaroudei* S. Bashiri & Abdollahz., sp. nov. (**Figure 9**).

MycoBank: MB850403.

#### 3.3.8 Diagnosis

*C. hedjaroudei* is phylogenetically close to *C. pistaciae* and *C. parapistaciae* but is morphologically differentiated by having distinct conidiophores that are absent and reduced to conidiogenous cells in both *C. pistaciae* and *C. parapistaciae*. Conidiomata with multiple internal locules are seen in *C. hedjaroudei*, while in both *C. pistaciae* and *C. parapistaciae*, conidiomata have a single internal locule. It is also differentiated from *C. pistaciae* by having significantly larger conidiogenous cells (12–25 × 2–5 µm vs. 7.1–8.9 × 1.1–1.5 µm) and slightly larger conidia (3.2–) 4–5 (–9.1) × (1–) 1.3–1.7 (–1.9) µm vs. (3.5–) 4–4.8 (–5.5) × (1.0–) 1.1–1.3 (–1.5) µm and from *C. parapistaciae* by having significantly larger conidiomata (800–2,800 µm vs. 335–590 µm), conidiogenous cells (12–25 × 2–5 µm vs. 7.6–9.6 × 1.2–1.6) and conidia (4–5 × 1.3–1.7 (av. = 4.5 × 1.5) µm vs. 3.5–4.3 × 0.9–1.1 µm).

#### 3.3.9 Etymology

Named after Dr. Ghorbanali Hedjaroude, emeritus professor of Tehran University, who significantly contributed to the knowledge of mycology in Iran.

#### 3.3.10 Type

IRAN, West Azarbaijan Province, Sardasht, from *Q. infectoria* branches, 36°18′15.8″ N/45°28′36.6″ E, 7 August 2016, S. Bashiri, IRAN 18257F (Holotype) (GenBank ITS: OR540617; *tef-1α*: OR561995; *tub2*: OR561904), ex-type IRAN 4325C = CBS 149769.

#### 3.3.11 Description

#### 3.3.12 Sexual morph

not observed.

#### 3.3.13 Asexual morph

Conidiomata on PDA pycnidial, globose, black-gray, covered by smoke gray mycelium, scattered, aggregated or solitary, with multiple internal locules, erumpent at maturity, exuding pale yellow or black conidial masses, 0.8–2.8 mm diameter (**Figures 9D–F**). Conidiophores hyaline, filamentous, branched or unbranched, thin walled, (4.9–) 7–22 (–25.5) µm (av. 14.2 ± 1.1 µm). Conidiogenous cells hyaline, smooth, cylindrical to subcylindrical, tapering to the apices, enteroblastic, phialidic with periclinal thickening, (8.5–) 12–25 (–33.8) × (1.5–) 2–5 (–2.8) µm (av. ± SD = 17.8 ± 1.3 × 2.2 ± 0.1 µm). Conidia hyaline, thin walled, smooth, eguttulate, allantoid, aseptate, (3.2–) 4–5 (–9.1) × (1–) 1.3–1.7 (–1.9) µm (av. ± SD = 4.5 ± 0.2 × 1.5 ± 0.1 µm) (**Figures 9G–J**).

#### 3.3.14 Culture characteristics

Colonies on PDA fast-growing, cottony, with dense aerial mycelium, smoke gray (21′′′′f) to olivaceous gray (21′′′′′i), margin smooth, reverse smoke gray (21′′′′f), reaching 90 mm diameter after 7 days at room temperature in the dark; on MEA cottony, with aerial mycelium, olivaceous buff (21′′′d) to greenish olivaceous (23′′′i), margin smooth, reverse olivaceous buff (21′′′d), reaching 90 mm diameter after 7 days at room temperature in the dark; on OA appressed, olivaceous buff (21′′′d) at the margin to greenish olivaceous (23′′′i), with some greenish olivaceous (23′′′i) to dull green (27′′m) sectors, reverse olivaceous buff (21′′′d), reaching 90 mm diameter after 7 days at room temperature in the dark (**Figures 9A–C**).

#### 3.3.15 Additional specimens examined

Iran, Kurdistan Province, Marivan, from *Q. libani* branches, 16 August 2016, S. Bashiri, IRAN 4842C = CJASB196 (35°35′57.4″ N/46°06′52.4″ E); Iran, Kurdistan Province, Marivan, from *Q. brantii* branches, 16 August 2016, S. Bashiri, CJASB197 (35°09′53.7″ N/46°30′69.9″ E); Iran, Kurdistan Province, Sarvabad, from *Q. brantii* branches, 16 August 2016, S. Bashiri, CJASB194 (35°09′87.1″ N/46°32′42.4″ E), CJASB195 (35°22′95.7″ N/46°12′89.4″ E); Iran, Kurdistan Province, Baneh, from *Q. libani* branches, 21 August 2016, S. Bashiri, CJASB190 (35°44′91.5″ N/45°49′74.0″ E), CJASB191 (22 August 2016, 36°08′19.9″ N/45°42′31.8″ E), CJASB193 (23 August 2016, 35°57′18.3″ N/46°00′58.5″ E); Iran, Kurdistan Province, Baneh, from *Q. brantii* branches, 23 August 2016, S. Bashiri, IRAN 4903C (35°50′76.5″ N/45°56′67.1″ E); Iran, West Azarbaijan Province, Sardasht, from *Q. infectoria* branches, 8 August 2016, S. Bashiri, CJASB184 (36°10′36.0″ N/45°30′15.3″ E); Iran, West Azarbaijan Province, Sardasht, from *Q. libani* branches, 8 August 2016, S. Bashiri, IRAN 4904C (36°07′23.4″ N/45°28′08.5″ E); Iran, Ilam Province, Eyvan, from *Q. brantii* branches, 15 September 2016, S. Bashiri, CJASB186/CJASB187 (33°50′84.8″ N/46°12′21.0″ E); Iran, Ilam Province, Sarableh, from *Q. brantii* branches, 15 September 2016, S. Bashiri, IRAN 4902C/CJASB189 (33°47′40.9″ N/46°30′93.1″ E); Iran, Kermanshah Province, Gilan-e-Gharb, from *Q. brantii* branches, 9 October 2016, S. Bashiri, CJASB199 (34°54′36.6″ N/46°32′65.6″ E), CJASB200 (15 September 2016, 34°05′02.3″ N/46°02′15.6″ E); Iran, Kermanshah Province, Paveh, from *Q. brantii* branches, 21 October 2016, S. Bashiri, CJASB201 (34°57′06.9″ N/46°27′80.5″ E); Iran, Kermanshah Province, Ravansar, from *Q. brantii* branches, 24 September 2016, S. Bashiri, CJASB198 (34°03′12.5″ N/46°27′19.3″ E); Iran, Lorestan Province, Chegini, from *Q. brantii* branches, 24 September 2016, S. Bashiri, CJASB202/CJASB203 (35°33′76.3″ N/46°06′18.8″ E); Iran, Lorestan Province, Poldokhtar, from *Q. brantii* branches, 27 September 2016, S. Bashiri, CJASB204 (33°18′12.2″ N/47°48′95.0″ E).

**Figure 10:**
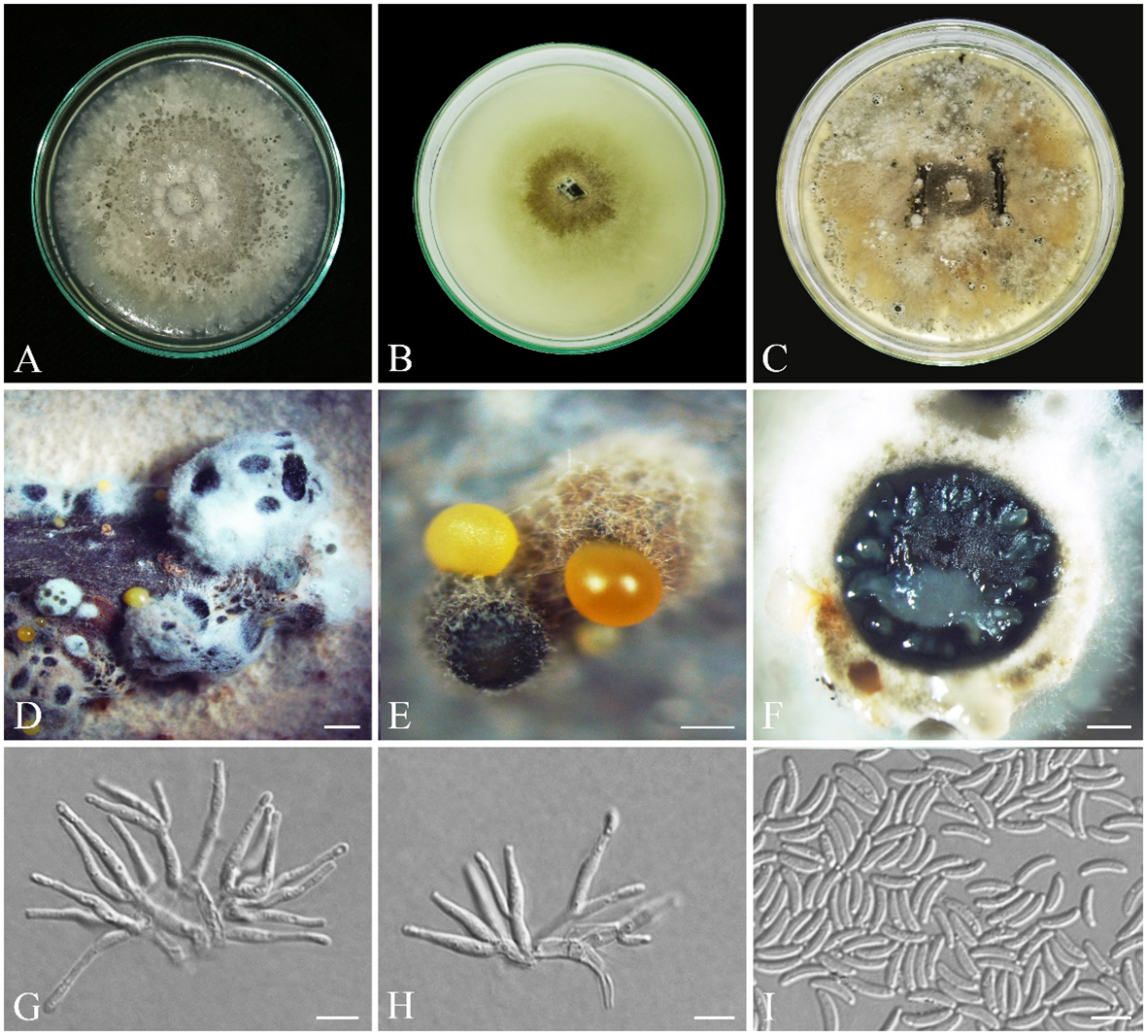
Seven-day-old colonies of *Cytospora zagrosensis* (IRAN 18256F, Holotype). **(A)** On PDA. **(B)** On OA. **(C)** Fourteen-day-old colonies on MEA at room temperature. **(D, E)** Conidiomata. **(F)** Cross-section of conidiomata. **(G, H)**. Conidiogenous cells. **(I)** Conidia. Scale bars: **(D)** 100 μm; **(E)** 600 μm; **(F)** 400 μm; **(G–I)** 5 μm.

*Cytospora zagrosensis* S. Bashiri & Abdollahz., sp. nov. (**Figure 10**).

MycoBank: MB850404.

#### 3.3.16 Diagnosis

*Cytospora zagrosensis* differs from closely related species *C. macropycnidia* by having smaller conidiomata (1.1–3.6 mm vs. 2.3–4.3 mm) and conidiogenous cells (av. = 12.8 × 2 µm vs. 17 × 2.5 μm) and long-narrow conidia (av. = 6.4 × 1.7 µm vs. 6 × 2.1 µm).

#### 3.3.17 Etymology

*zagrosensis* refers to the Zagros forests located at the western part of Iran, from which the fungus was first collected.

#### 3.3.18 Type

IRAN, Kurdistan Province, Baneh, from *Q. libani* branches, 35°18′81.1″ N/46°21′89.2″ E, 22 August 2016, S. Bashiri, IRAN 18256F (Holotype) (GenBank LSU: OR540615; ITS: OR540618; *rpb2*: OR540303; *tef-1α*: OR5611993; *tub2*: OR561905; *act1*: OR550646), ex-type IRAN 4324C = CBS 149765.

#### 3.3.19 Description

#### 3.3.20 Sexual morph

not observed.

#### 3.3.21 Asexual morph

Conidiomata on PDA pycnidial, brown to black, covered by buff to white mycelium, aggregated or solitary, with multiple internal locules, erumpent at maturity, exuding yellow or pale orange conidial masses, 1.1–3.6 mm diameter (**Figures 10D–F**). Conidiophores hyaline, thin walled, reduced to short discrete basal cells with whorls of two to three concentric conidiogenous cells on the tips. Conidiogenous cells hyaline, smooth, subcylindrical, tapering to the apices, enteroblastic, phialidic with periclinal thickening, (6.1–) 10–16 (–19.5) × (1.4–) 1.7–2.3 (–2.5) μm (av. ± SD = 12.8 ± 0.4 × 2 ± 0.1 µm). Conidia hyaline, thin walled, smooth, eguttulate, allantoid, aseptate, (5–) 5.5–7 (–8) × (1.3–) 1.4–1.9 (–2) μm (av. ± SD = 6.4 ± 0.1 × 1.7 ± 0.1 µm) (**Figures 10G–I**).

#### 3.3.22 Culture characteristics

Colonies on PDA cottony, olivaceous buff (21′′′d) to smoke gray (21′′′′f), margin slightly irregular or lobate, reverse pale luteous (17d) to luteous (17i), reaching 80 mm diameter after 7 days at room temperature in the dark, on MEA cottony, olivaceous buff (21′′′d) to smoke gray (21′′′′f), reverse buff (21′′′d) to whitish, margin smooth, reaching 50 mm diameter after 7 days at room temperature in the dark; on OA appressed, olivaceous buff (21′′′d) to greenish olivaceous (23′′′i), margin smooth, reverse olivaceous buff (21′′′d), reaching 60 mm diameter after 7 days at room temperature in the dark (**Figures 10A–C**).

#### 3.3.23 Additional specimens examined

Iran, Kermanshah Province, Paveh, from *Q. brantii* branches, 21 October 2016, S. Bashiri, IRAN 4326C = CBS 149767 (GenBank LSU: OR540616; ITS: OR540619; *rpb2*: OR540304; *tef-1α*: OR561994; *tub2*: OR561906; *act1*: OR550647) (34°57′06.9″ N/46°27′80.5″ E), CJASB165/CJASB166/CJASB177/CJASB178 (34°57′06.9″ N/46°27′80.5″ E); Iran, Kermanshah Province, Javanrud, from *Q. brantii* branches, 21 October 2016, S. Bashiri, CJASB167 (34°49′26.8″ N/46°30′15.1″ E); Iran, Kermanshah Province, Gilan-e-Gharb, from *Q. brantii* branches, 9 October 2016, S. Bashiri, IRAN 4838C = CJASB179/CJASB169 (34°54′36.6″ N/46°32′65.6″ E), CJASB168/CJASB180 (15 September 2016, 34°05′02.3″ N/46°02′15.6″ E); Iran, Kermanshah Province, Eslamabad-e-Gharb, from *Q. brantii* branches, 10 October 2016, S. Bashiri, CJASB163 (33°47′87.6″N/46°52′11.4″ E), CJASB164 (11 October 2016, 33°58′68.9″ N/46°29′92.0″ E); Iran, Kurdistan Province, Marivan, from *Q. libani* branches, 16 August 2016, S. Bashiri, IRAN 4839C = CJASB161 (35°24′97.6″ N/46°16′08.5″ E), CJASB176 (35°38′57.4″ N/46°06′52.4″ E); Iran, Kurdistan Province, Marivan, from *Q. brantii* branches, 15 August 2016, S. Bashiri, CJASB162 (35°24′78.4″ N/46°10′73.4″ E); Iran, Kurdistan Province, Marivan, from *Q. infectoria* branches, 28 July 2016, S. Bashiri, CJASB175 (35°28′83.4″ N/46°06′22.8″ E); Iran, Kurdistan Province, Sarvabad, from *Q. libani* branches, 15 August 2016, S. Bashiri, CJASB160 (35°22′90.1″ N/46°09′28.3″ E), CJASB174 (35°23′86.3″ N/46°12′49.4″ E); Iran, Lorestan Province, Chegini, from *Q. brantii* branches, 26 September 2016, S. Bashiri, CJASB170 (33°28′01.4″ N/48°00′43.3″ E), CJASB182 (27 September 2016, 33°32′49.7″ N/48°05′23.8″ E); Iran, Lorestan Province, Poldokhtar, from *Q. brantii* branches, 27 September 2016, S. Bashiri, IRAN 4901C (33°18′12.2″ N/47°48′95.0″ E), CJASB181 (33°14′33.1″ N/47°39′64.6″ E); Iran, Lorestan Province, Kuhdasht, from *Q. brantii* branches, 27 September 2016, S. Bashiri, CJASB172 (33°31′28.4″ N/47°47′.246″ E).

**Figure 11:**
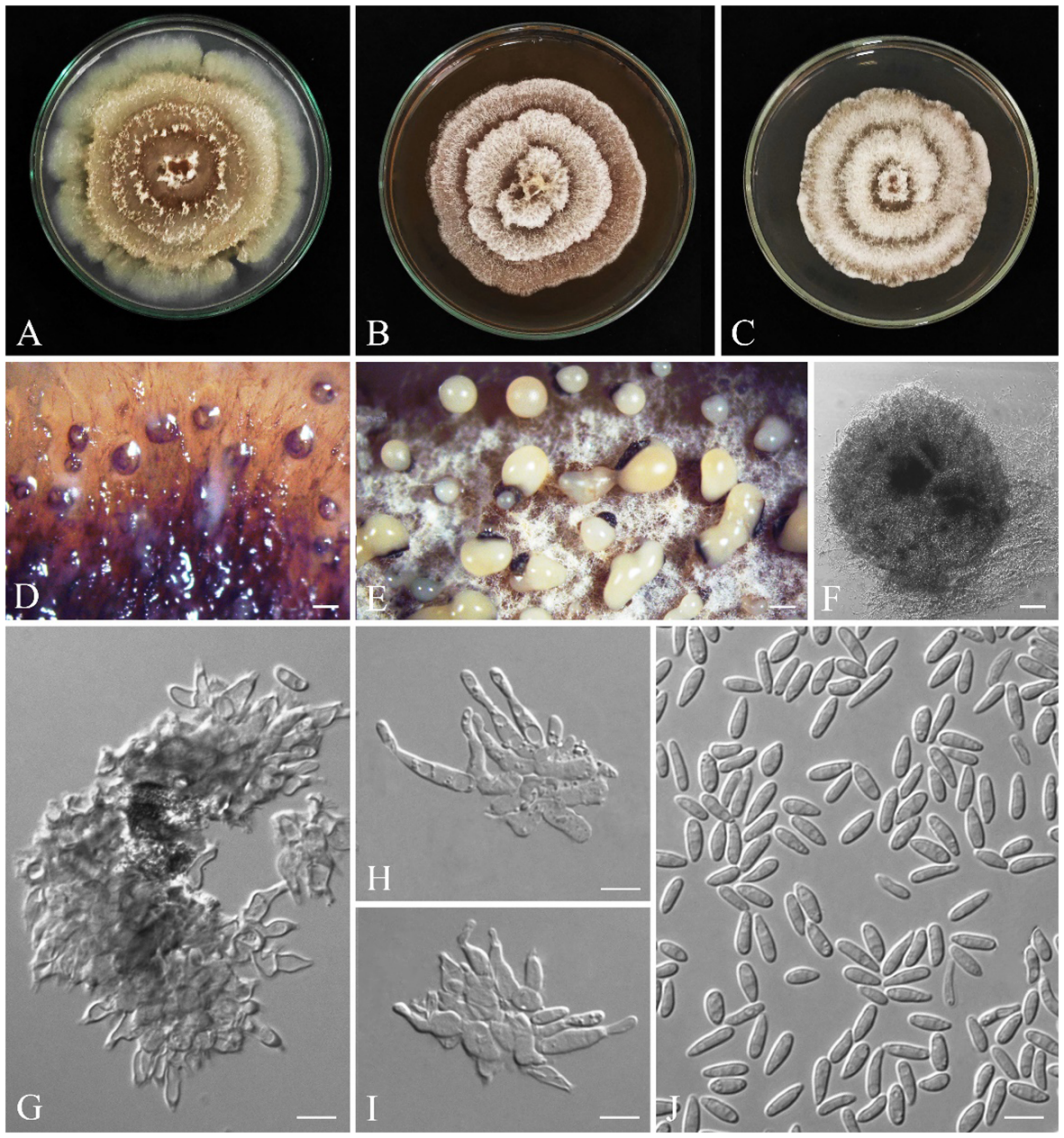
Seven-day-old colonies of *Gnomoniopsis quercicola* (IRAN 18252F, Holotype). **(A)** On PDA. **(B)** On MEA. **(C)** On YMA at room temperature. **(D–F)** Conidiomata. **(G–I)** Conidiogenous cells. **(J)** Conidia. Scale bars: **(D, E)** 200; **(F)** 25 μm; **(G–J)** 5 μm.

*Gnomoniopsis quercicola* S. Bashiri & Abdollahz., sp. nov. (**Figure 11**).

MycoBank: MB850402.

#### 3.3.24 Diagnosis

Generally, it is difficult to discriminate all *Gnomoniopsis* species based on morphology. *Gnomoniopsis quercicola* clusters with *G. paraclavulata* in a distinct clade. It is discriminated from *G. paraclavulata* by having smaller conidia (6.2 ± 0.1 × 2.2 ± 0.04 µm vs. 7.5 ± 0.5 × 3 ± 0.3 μm) and a faster growing rate (on PDA: 90 mm/9 d vs. 90 mm/40 d; on MEA and MYA: 90/14 d vs. 90 mm/40 d).

#### 3.3.25 Etymology

*quercicola* refers to *Quercus*, the host genus from which the fungus was first isolated.

#### 3.3.26 Type

IRAN, Kurdistan Province, Baneh, from *Q. brantii* branches, 36°07′17.7″ N/45°41′37.0″ E, 22 August 2016, S. Bashiri, IRAN 18252F (Holotype) (GenBank ITS: OR540614; *tef-1α*: OR561996 *tub2*: OR561907), ex-type IRAN 4313C = CBS 149773.

#### 3.3.27 Description

#### 3.3.28 Sexual morph

not observed.

#### 3.3.29 Asexual morph

Conidiomata on PDA aggregated or solitary, globose, without neck, semi-immersed, dark yellow to brown, erumpent at maturity, exuding pale yellow slimy conidial mass after 14 days at room temperature (**Figures 11D–F**). Conidiophores are indistinct, often reduced to conidiogenous cells. Conidiogenous cells cylindrical to lageniform, tapering toward apex, hyaline, phialidic with periclinal thickening, (4.3–) 5–12 (–13.9) × (1.6–) 2–3 (–4.5) µm (av. ± SD = 8.5 ± 0.4 × 2.5 ± 0.1 µm). Conidia oval to oblong, hyaline, smooth, often straight, rarely slightly curved, base subtruncate to bluntly rounded, (4.5–) 5–7 (–8.3) × (1.3–) 1.9–2.5 (–2.8) µm (av. ± SD = 6.2 ± 0.1 × 2.2 ± 0.04 µm) (**Figures 11G–J**).

#### 3.3.30 Culture characteristics

Colonies on PDA cottony, with aerial mycelium, colony margin forming a concentric ring with sparse aerial mycelium, followed by additional rings, creating a lobed rosette-like appearance, buff (21′′′d) to olivaceous (19′′k) at the center, olivaceous buff (21′′′d) to greenish olivaceous (23′′′i), reaching 90 mm after 9 days at room temperature in the dark; on MEA cottony, with aerial mycelium, spreading out in concentric rings, creating a lobed rosette-like appearance, buff (21′′′d) to whitish, reaching 90 mm after 14 days at room temperature in the dark; on MYA cottony, with aerial mycelium, spreading out in concentric rings, creating a lobed rosette-like appearance, buff (21′′′d) to whitish, reaching 90 mm after 14 days at room temperature in the dark (**Figures 11A–C**).

#### 3.3.31 Additional specimens examined

Iran, Kurdistan Province, Baneh, from *Q. libani* branches, 22 August 2016, S. Bashiri, IRAN 4875 = CBS 149774/CJASB296 (36°05′01.8″ N/45°40′32.8″ E), CJASB298 (23 August 2016, 35°52′30.8″ N/45°59′19.6″ E); Iran, Kurdistan Province, Baneh, from *Q. brantii* branches, 23 August 2016, S. Bashiri, CJASB299 (35°52′.308″ N/45°59′.196″ E), CJASB294 (23 August 2016, 36°07′17.7″ N/45°41′37.0″ E); Iran, Kurdistan Province, Sarvabad, from *Q. libani *branches, 15 August 2016, S. Bashiri, CJASB300 (35°22′90.1″ N/46°09′28.3″ E), CJASB302(16 August 2016, 35°23′86.3″ N/46°12′49.4″ E)/CJASB303 (16 August 2016, 35°05′64.3″ N/46°35′30.9″ E); Iran, Kurdistan Province, Sarvabad, from *Q. brantii* branches, 16 August 2016, S. Bashiri, CJASB304 (35°06′79.9″ N/46°32′58.1″ E); Iran, Kurdistan Province, Sarvabad, from *Q. infectoria* branches, 15 August 2016, S. Bashiri, CJASB301 35°22′95.7″ N/46°12′89.5″ E; Iran, West Azarbaijan Province, Sardasht, from *Q. libani* branches, 8 August 2016, S. Bashiri, CJASB283/IRAN 4905C (36°06′07.2″ N/45°29′64.5″ E), CJASB286 (36°10′20.1″ N/45°29′12.3″ E); Iran, West Azarbaijan Province, Sardasht, from *Q. infectoria* branches, 8 August 2016, S. Bashiri, CJASB285 (36°07′23.4″ N/45°28′08.5″ E), CJASB286 (36°10′20.1″ N/45°29′12.3″ E); Iran, West Azarbaijan Province, Piranshahr, from *Q. infectoria* branches, 7 August 2016, S. Bashiri, CJASB287/CJASB288 (36°23′12.2″ N/45°23′33.9″ E), CJASB286 (36°10′20.1″ N/45°29′12.3″ E); Iran, Kermanshah Province, Ravansar, from *Q. brantii* branches, 21 October 2016, S. Bashiri, CJASB305/CJASB306 (34°03′12.5″ N/46°27′19.3″ E); Iran, Kermanshah Province, Paveh, from *Q. brantii* branches, 21 October 2016, S. Bashiri, IRAN 4907C/CJASB308/CJASB309 (34°57′06.9″ N/46°27′80.5″ E); Iran, Ilam Province, Ilam, from *Q. brantii* branches, 13 September 2016, S. Bashiri, CJASB289/CJASB290 (33°42′15.6″ N/46°22′63.5″ E), CJASB291 (33°35′67.3″ N/46°26′50.6″ E); Iran, Ilam Province, Eyvan, from *Q. brantii* branches, 13 September 2016, S. Bashiri, IRAN 4906C/CJASB293 (33°50′84.8″ N/46°12′21.0″ E).

### 3.4 Pathogenicity tests

**Figure 12:**
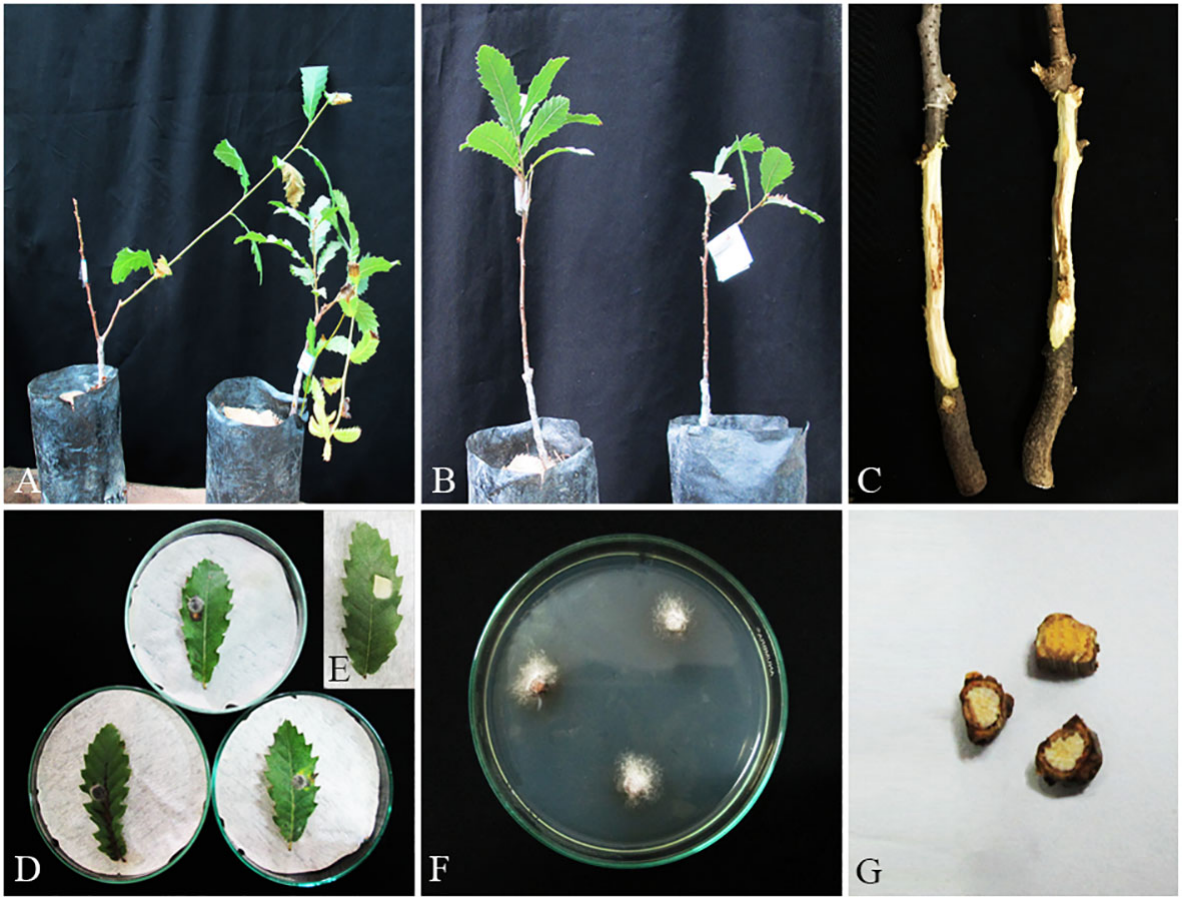
Pathogenicity tests and disease symptoms caused by *A. iranensis* on oak seedlings in greenhouse **(A–C, G)** and leaves under *in vitro* **(D, E)** conditions. **(A)** Inoculated plants after 2 months. **(B)** Control. **(C)** Necrotic lesion on stems. **(D, E)** Necrotic spot on leaves and control. **(F)** *A*. *iranensis* colony re-isolated from inoculated seedlings. **(G)** Stem cross-sections showing wood necrosis and discoloration.

**Figure 13:**
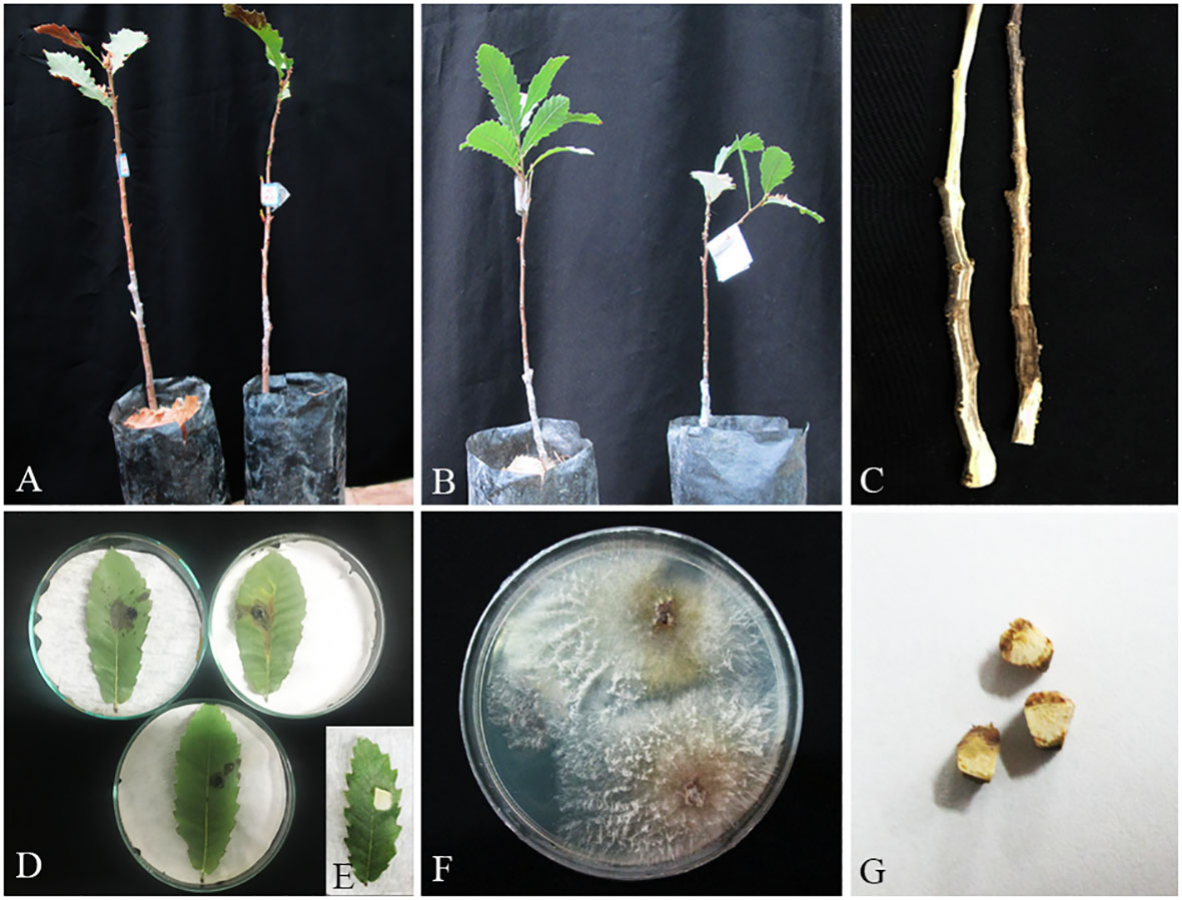
Pathogenicity tests and disease symptoms caused by *C. hedjaroudei* on oak seedlings in greenhouse **(A–C, G)** and leaves under *in vitro* **(D, E)** conditions. **(A)** Inoculated plants after 2 months. **(B)** Control. **(C)** Necrotic lesion on stems. **(D, E)** Necrotic spot on leaves and control. **(F)** *C. hedjaroudei* colony re-isolated from inoculated seedlings. **(G)** Stem cross-sections showing wood necrosis and discoloration.

**Figure 14:**
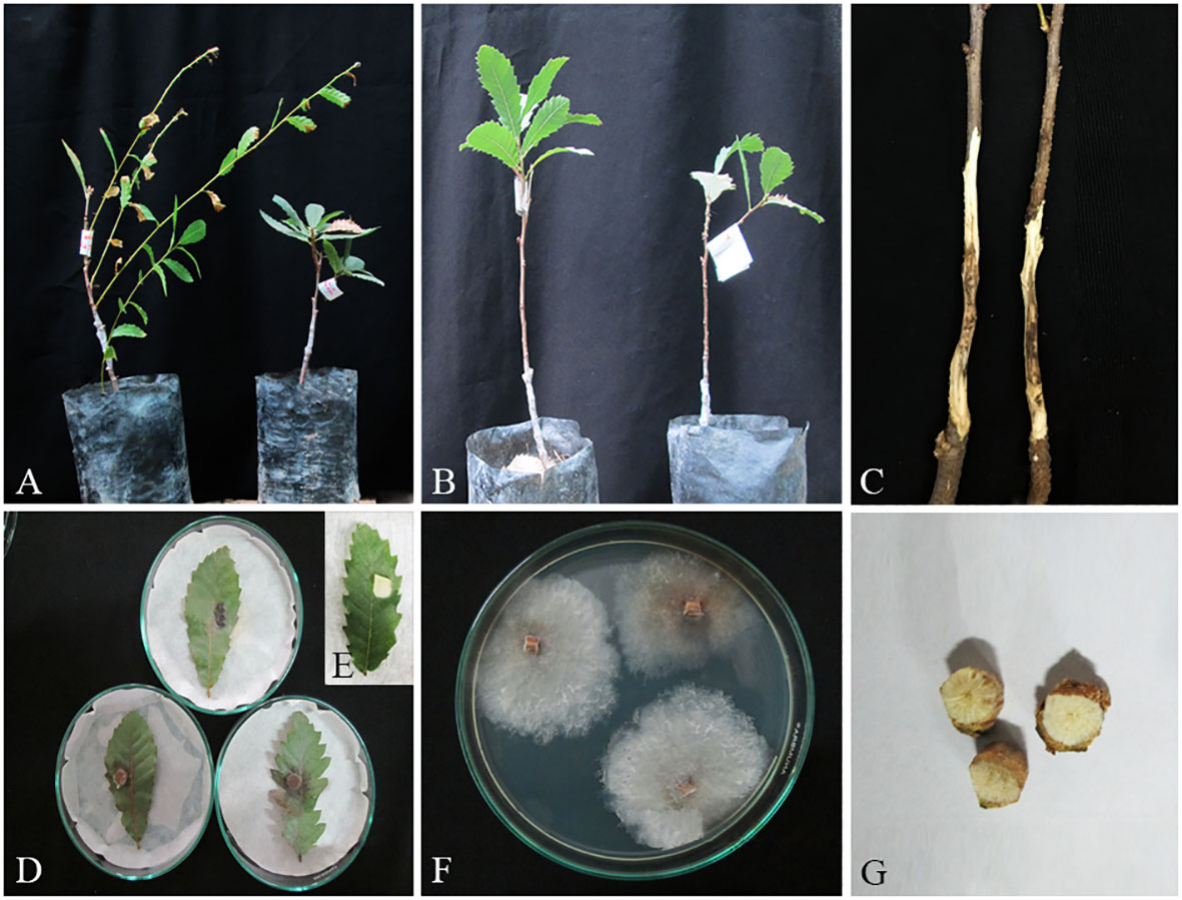
Pathogenicity tests and disease symptoms caused by *C. zagrosensis* on oak seedlings in greenhouse **(A–C, G)** and leaves under *in vitro* **(D,E)** conditions. **(A)** Inoculated plants after 2 months. **(B)** Control. **(C)** Necrotic lesion on stems. **(D, E)** Necrotic spot on leaves and control. **(F)** *C. zagrosensis* colony re-isolated from inoculated seedlings. **(G)** Stem cross-sections showing wood necrosis and discoloration.

**Figure 15:**
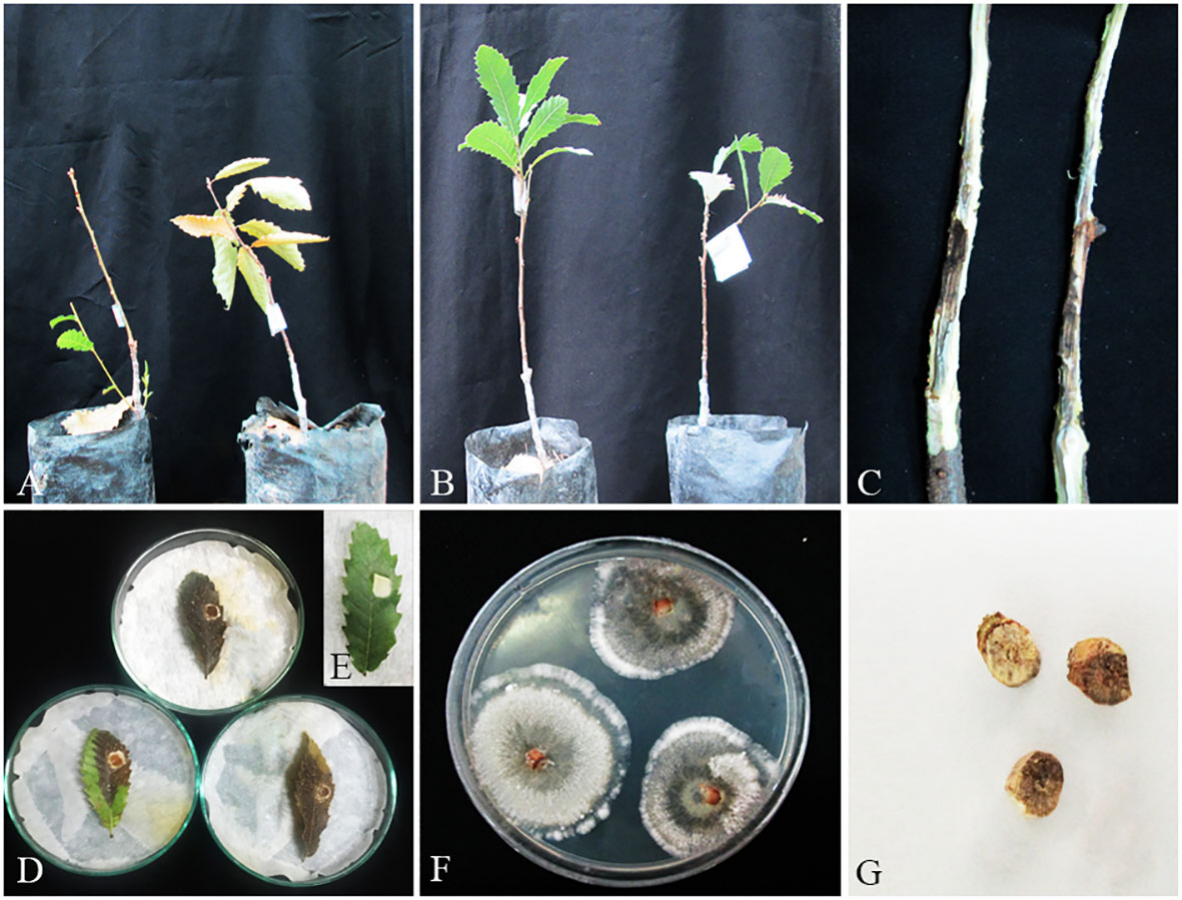
Pathogenicity tests and disease symptoms caused by *G*. *quercicola* on oak seedlings in greenhouse **(A–C, G)** and leaves under *in vitro* **(D,E)** conditions. **(A)** Inoculated plants after 1 month. **(B)** Control. **(C)** Necrotic lesion on stems. **(D, E)** Necrotic spot on leaves and control. **(F)** *G*. *quercicola* colony re-isolated from inoculated seedlings. **(G)** Stem cross-sections showing wood necrosis and discoloration.

In this survey, pathogenicity of representative isolates was examined under *in vitro* (on leaves) and greenhouse (on seedling stems) conditions (**Figures 12**–**15**, **Supplementary Figures 1–4**). In both experiments, 19 fungal species were determined as pathogenic, and *Alternaria* spp. (*A. alternata*, *A. atra*, and *A. contlous*), *Chaetomium globosum*, and *Parachaetomium perlucidum* were nonpathogenic.

In the phytotoxicity assay under *in vitro* conditions on oak leaves, we qualitatively evaluated and grouped pathogenic species in four classes as highly virulent (*Biscogniauxia persica*, *B. rosacearum*, *Botryosphaeria dothidea*, *Gnomoniopsis quercicola*, and *Neoscytalidium dimidiatum*), moderately virulent (*Cosmospora butyri*, *Didymella glomerata*, *Fusarium anulatum*, *Kalmusia variispora*, and *Neocosmospora* sp.), weakly virulent (*Alloeutypa iranensis*, *Cytospora hedjaroudei*, *C. zagrosensis*, *Fimetariella rabenhorstii*, *Neocosmospora metavorans*, *Phaeoacremonium tuscanicum*, and *Stilbocrea anihashemiana*), and very weak virulent (*Apiospora intestini* and *Nigrospora* sp.).

In pathogenicity tests under greenhouse conditions, *Biscogniauxia rosacearum*, *B. persica*, *Botryosphaeria dothidea*, *G. quercicola*, and *N. dimidiatum* were the most virulent species, which caused a quick decline and death of inoculated seedlings after 28–30 days. Thus, to evaluate the pathogenicity of these species in a distinct analysis, we recorded the external and internal symptoms and analyzed them after 30 days (**Table 2**). Pathogenicity of the other examined species were recorded after 60 days and analyzed separately (**Table 3**). In the first statistical analyses, significant differences were determined in lesion lengths between species (*F* = 54.22, *p* < 0.001) and *B. rosacearum* was the most virulent species and *G. quercicola* was the most virulent coelomycetous species. *Gnomoniopsis quercicola* showed high phytotoxic activity on leaves examined under *in vitro* conditions (**Figure 15D**). Severe leaf yellowing and defoliation on inoculated seedlings and extended brown wood necrosis in cross-sections of inoculated stems were observed 28–30 days after inoculation under greenhouse conditions (**Figure 15**). *Botryosphaeria dothidea* and *N. dimidiatum* showed high phytotoxic activity on leaves (**Supplementary Figures 1–4D**) and inoculated seedlings showed severe leaf blight and defoliation together with wedge-shaped and irregular wood necrosis in cross-sections of inoculated stems 28–30 days after inoculation (**Supplementary Figures 1–4**). The second statistical analysis also indicated significant differences in lesion lengths between species (*F* = 318.33, *p* < 0.001) and all coelomycetous species; *A. iranensis*, *C. hedjaroudei*, *C. zagrosensis*, *D. glomerata*, and *K. variispora* were pathogenic and caused leaf necrosis, blight, and defoliation on oak seedlings under greenhouse conditions 60 days after inoculation. Moreover, cross-sections of inoculated stems showed an intermediate vascular discoloration and irregular wood necrosis (**Table 3**, **Figures 12**–**14**, **Supplementary Figures 2, 3**). All inoculated pathogens were re-isolated from inoculated leaves and seedling stems, confirming Koch’s postulates (**Figures 12**–**15**, **Supplementary Figures 1–4**).

**Table 2:** Number of isolates, observed symptoms, and mean lesion length measured in pathogenicity studies of five highly virulent species recorded and analyzed after 30 days.

Species	Isolates number	External symptoms	Internal symptoms	Lesion length (mm) ± SD
*Biscogniauxia rosacearum*	10	Chronic blight, defoliation, gummosis	Irregular necrosis	12.05 ± 0.3
*Gnomoniopsis quercicola*	2	Chronic blight, defoliation	Irregular necrosis	11.50 ± 0.2
*Botryosphaeria dothidea*	3	Chronic blight, defoliation	Wedge shaped necrosis	10.85 ± 0.19
*Neoscytalidium dimidiatum*	2	Blight	Irregular necrosis	10.83 ± 0.28
*Biscogniauxia persica*	2	Chronic blight, defoliation	Wedge shaped necrosis	10.77 ± 0.21
Control	–	No symptom	No symptom	2.06 ± 0.12
LSD value at α = 0.05	–	–	–	0.24

**Table 3:** Number of isolates, observed symptoms, and mean lesion length measured in pathogenicity studies of 19 species recorded and analyzed after 60 days.

Species	Isolate number	External symptoms	Internal symptoms	Lesion length (mm) ± SD
*Neocosmospora* sp.	4	Leaf necrosis	Irregular necrosis	10.66 ± 0.26
*N. metavorans*	2	Leaf necrosis	Irregular necrosis	10.30 ± 0.24
*Fusarium annulatum*	2	Leaf necrosis, defoliation	Irregular necrosis	9.36 ± 0.18
*Didymella glomerata*	2	Leaf necrosis, defoliation, blight	Irregular necrosis	9.16 ± 0.18
*Phaeoacremonium tuscanicum*	2	Leaf necrosis	Irregular necrosis	8.30 ± 0.32
*Cytospora hedjaroudei*	2	Leaf necrosis	Irregular necrosis	8.26 ± 0.25
*Cytospora zagrosensis*	2	Leaf necrosis	Irregular necrosis	7.93 ± 0.16
*Kalmusia variispora*	4	Leaf necrosis	Irregular necrosis	7.80 ± 0.25
*Stilbocrea banihashemiana*	2	Defoliation, blight	Irregular necrosis	7.80 ± 0.2
*Alloeutypa iranensis*	2	Leaf necrosis	Irregular necrosis	7.41 ± 0.28
*Cosmospora butyri*	2	Leaf necrosis	Irregular necrosis	7.16 ± 0.21
*Fimetariella rabenhorstii*	3	Leaf necrosis	Weak irregular necrosis	5.14 ± 0.2
*Nigrospora* sp.	1	Weak leaf necrosis	Weak irregular necrosis	4.26 ± 0.25
*Apiospora intestini*	1	Weak leaf necrosis	Weak irregular necrosis	4.06 ± 0.11
*Parachaetomium perlucidum*	1	No symptoms	No symptoms	2.36 ± 0.32
*Chaetomium globosum*	3	No symptoms	No symptoms	2.31 ± 0.25
*Alternaria alternata*	1	No symptoms	No symptoms	2.26 ± 0.05
*A. atra*	1	No symptoms	No symptoms	2.16 ± 0.15
*A. cantlous*	1	No symptoms	No symptoms	2.13 ± 0.05
Control	–	No symptoms	No symptoms	2.06 ± 0.11
LSD value at α = 0.05	–	–	–	0.3

## 4 Discussion

In this study, we focused on oak decline, a growing threat to Zagros forests, and collected a large fungal collection from oak trees showing various types of external and internal disease symptoms in Zagros forests located in five western provinces: Ilam, Kermanshah, Kurdistan, Lorestan, and West Azarbaijan. Generally, depending on a variety of biotic and abiotic factors (e.g., host plant, microbial communities, and climate factors), management and exploitation systems, method and time of sampling, and isolation procedure in a study composition, the frequency and diversity of fungal species associated with declined woody plants vary in different geographic regions around the world (Lynch et al., 2013; Linaldeddu et al., 2016; Hashemi et al., 2017; Alidadi et al., 2018; Ghobad-Nejhad et al., 2018; Alidadi et al., 2019; Pinna et al., 2019; Mahamedi et al., 2020; Bahmani et al., 2021; Nahvi Moghadam et al., 2022). Based on DNA sequence data and morphological features, we characterized 24 fungal species belonging to 19 genera from 10 different families, including *Apiosporaceae* (*Apiospora* and *Nigrospora*), *Bionecteriaceae* (*Stilbocrea*), *Bombardiaceae* (*Fimetariella*), *Botryosphaeriaceae* (*Botryosphaeria* and *Neoscytalidium*), *Chaetomiaceae* (*Chaetomium*, *Parachaetomium*), *Cytosporaceae* (*Cytospora*), *Diatrypaceae* (*Alloeutypa*), *Didymellaceae* (*Didymella*), *Didymosphaeriaceae* (*Kalmusia*), *Gnomoniaceae* (*Gnomoniopsis*), *Graphostromataceae* (*Biscogniauxia*), *Nectriaceae* (*Cosmospora*, *Fusarium*, and *Neocosmospora*), *Pleosporaceae* (*Alternaria*), and *Togninaceae* (*Phaeoacremonium*). In this paper, we have listed and provided frequency and distribution of the identified fungi at the genus and species levels and focused on phylogeny and pathology of coelomycetous fungi, a well-known morphological group in fungal pathogens.

*Biscogniauxia* (*B. rosacearum* and *B. persica*), *Neocosmospora* (*N. metavorans* and *Neocosmospora* sp.), and *Cytospora* (*C. hedjaroudei* and *C. zagrosensis*) were the most prevalent identified fungi associated with oak decline. Among the coelomycetous fungi, members of two well-known families *Botryosphaeriaceae* (*Botryosphaeria* and *Neoscytalidium*) and *Cytosporaceae* (*Cytospora*) in association with decline of woody plants were isolated from declined oak trees in the central and northern part of Zagros forests.

*Botryosphaeriaceae* members are common fungal pathogens, endophytes, or saprobes on woody plants (Phillips et al., 2013; Moricca et al., 2016; Kazemzadeh Chakusary et al., 2019; Mahamedi et al., 2020; Brazee et al., 2023). *Botryosphaeria* and *Neoscytalidium* species are among the most virulent members of *Botryosphaeriaceae* associated with canker, dieback, fruit rot, and decline symptoms in a broad spectrum of woody plants (including *Quercus* spp.) (Luque et al., 2000; Sánchez et al., 2003; Linaldeddu et al., 2009; Marsberg et al., 2017; Kazemzadeh Chakusary et al., 2019; Mahamedi et al., 2020; Derviş and Özer, 2023). A search of Index Fungorum (November 2023; www.indexfungorum.org) lists more than 200 *Botryosphaeria* species in which DNA sequence data are available for some 20 species. Thus far, five *Neoscytalidium* species, namely, *N. dimidiatum*, *N. hylocereum*, *N. novaehollandiae*, *N. oculi*, and *N. orchidacearum*, have been described (November 2023; www.indexfungorum.org). Of these, *N. novaehollandiae* and *N. orchidacearum* have been reduced to synonymy with *N. dimidiatum* (Zhang et al., 2021). *Botryosphaeria dothidea* has previously been isolated as saprophyte from pycnidia on dead twigs of an unknown *Quercus* species in Gardane-Heyran, north of the country (Abdollahzadeh, 2009; Abdollahzadeh et al., 2013), but it is reported here for the first time as a pathogenic species isolated from necrotic wood tissues of oak trees in Iran. We isolated this species from all three oak species in Ilam, Kermanshah, Kurdistan, Lorestan, and West Azarbaijan provinces, while *N. dimidiatum* was found only from *Q. brantii* in Kermanshah, Ilam, and Lorestan provinces with a higher mean annual temperature. *Neoscytalidium dimidiatum* has already been reported as an endophytic (Ghasemi-Esfahlan et al., 2019) or pathogenic fungal species (Alidadi et al., 2019; Sabernasab et al., 2019) associated with Persian oak (*Q. brantii*) in Zagros forests located in Kermanshah, Ilam, and Lorestan provinces, but it is reported for the first time from Kurdistan and West Azarbaijan provinces. In this study, *B. dothidea* and *N. dimidiatum* were determined as highly virulent in both pathogenicity experiments on leaves and seedling stems of *Q. brantii*. Pathogenicity of both species on oak has previously been confirmed (Sánchez et al., 2003; Turco et al., 2006; Alidadi et al., 2019; Sabernasab et al., 2019).

Members of diaporthalean fungi are associated with several diseases including canker and dieback in economically and ecologically important woody plants such as *Quercus* species (Luque et al., 2000; Lynch et al., 2014; Fan et al., 2018; Jiang et al., 2018, 2019; Zhu et al., 2019, 2021). Among the diaporthalean fungi, *Cytospora* with approximately 700 species listed in Index Fungorum (November 2023; www.indexfungorum.org) is the most common and widespread genus associated with a wide variety of woody plants around the world, which causes various disease symptoms such as canker and dieback or found as endophyte and saprobe (Adams et al., 2005; Lawrence et al., 2018; Azizi et al., 2020; Fan et al., 2020; Hanifeh et al., 2022; Ilyukhin et al., 2023). *Cytospora* species are found in association with canker and dieback diseases on various *Quercus* species (Kowalski, 1991; Adams et al., 2005; Lynch et al., 2014; Fan et al., 2018; Lawrence et al., 2018; Pinna et al., 2019; Pan et al., 2021). Very few *Cytospora* species have been reported from oak trees (*Q. brantii*) in Iran including *C. intermedia* from Fars Province (Fotouhifar et al., 2008), *Cystospora* sp.1 and *Cytospora* sp.2 from Kohgiluyeh and Boyer-Ahmad (Ghobad-Nejhad et al., 2017, 2018), and *C. ribis* from Ilam (Alidadi et al., 2019) provinces. In this study, based on morphology and phylogenetic analyses of a dataset combining sequences of five loci LSU, ITS, *rpb2*, *ef-1α*, *tub2*, and *act1*, we characterized two new species named *C. hedjaroudei* sp. nov. and *C. zagrozensis* sp. nov. isolated from all three *Quercus* species. *Cytospora zagrosensis* isolates were collected from Kermanshah, Kurdistan, and Lorestan provinces while *C. hedjaroudei* was also collected from West Azarbaijan and Ilam provinces. Thus far, some 15 *Cytospora* species have been reported from oak trees including the most known or recently described species: *Cytospora chrysosperma*, *C. quercicola*, *C. quercinum*, *C. prunicola*, *C. pubescentis*, and *C. vinacea* (Preston, 1945; Spaulding, 1961; Senanayake et al., 2017; Shang et al., 2020, 2020; Pan et al., 2021). *Cytospora hedjaroudei* and *C. zagrosensis* can be distinguished from closely related species-based morphology and DNA sequenced data. In pathogenicity experiments on oak leaves and seedling stems, both *Cytospora* species were recognized as weakly virulent.

*Gnomoniaceae*, as the second largest family of *Diaporthales*, is found on leaves and twigs of hardwood trees, shrubs, and herbaceous plants (Walker et al., 2010). *Gnomoniopsis* Berl., as a distinct genus based on the type species *Gnomoniopsis chamaemori* (Fr.) Berl., is isolated only from three plant families *Fagaceae*, *Onagraceae*, and *Rosaceae* (Wang et al., 2022). Thus far, several species, namely, *G. clavulata*, *G. paraclavulata*, *G. daii*, *G. fagacearum*, and *G. silvicola*, have been recorded on various *Quercus* species (Sogonov et al., 2008; Walker et al., 2010; Jiang et al., 2021; Jankowiak et al., 2022). To date, *Discula quercina* is the only *Gnomoniaceae* species reported from *Q. infectoria*, *Q. macranthera*, and *Q. rubra* in Iran (Khabiri, 1958; Ghasemi-Esfahlan et al., 2019; Hanifeh et al., 2019). In this study, we characterized a new *Gnomoniopsis* species named *Gnomoniopsis quercicola* sp. nov. from all three *Quercus* species in Zagros forests of West Azarbaijan, Kurdistan, Kermanshah, and Ilam provinces. *Gnomoniopsis quercicola* was placed in a distinct clade close to *G. paraclavulata* with significant differences in DNA sequence data and growth rate of colony on culture media. This species was the most pathogenic coelomycetous species and the second highly virulent species after *B. rosacearum* in both pathogenicity experiments on oak leaves and seedling stems.

*Diatrypaceae* family members are other fungal pathogens that have been isolated in association with canker and dieback diseases of a broad spectrum of woody hosts (Acero et al., 2004; Trouillas and Gubler, 2010; Trouillas et al., 2011, 2011; Mehrabi et al., 2015; Mehrabi et al., 2016; Mehrabi et al., 2019; Zhu et al., 2021). Several *Diatrypaceae* members belonging to various genera (e.g., *Alloeutypa*, *Cryptovalsa*, *Diatrype*, *Diatrypella*, *Eutypa*, *Eutypella*, and *Libertella*) have been isolated from oak trees (Acero et al., 2004; Mehrabi and Hemmati, 2013; Mehrabi et al., 2015; Mehrabi et al., 2016; Mehrabi et al., 2019; Zhu et al., 2021). Among these genera, *Alloeutypa* has recently been introduced to encompass a new species, *A. milinensis* (type species), and a new combination, *Alloeutypa flavovirens* (Ma et al., 2023). In this study, we recognized a new species in *Alloeutypa* close to *A. flavovirens* named *Alloeutypa iranensis* sp. nov. Based on morphology and DNA sequence data, this species is obviously different from *A. flavovirens* and *A. milinensis*. In pathogenicity experiments, *A. iraniensis* was weakly virulent on oak leaves and seedling stems.

*Didymella glomerata* (*Didymellaceae*) and *K. variispora* (*Didymosphaeriaceae* = *Montagnulaceae*), two phoma-like fungal pathogens, are seen among the pathogenic fungi associated with woody plants showing canker, dieback, and decline symptoms or as endophyte and saprobe (Holz et al., 1989; Thomidis et al., 2011; Verkley et al., 2014; Alidadi et al., 2018, 2019; Ghobad-Nejhad et al., 2017; Bahmani et al., 2021; Nahvi Moghadam et al., 2022). Recently, *D. glomerata* was isolated from *Q. brantii* in Zagros forests of Ilam as endophyte and pathogen (Alidadi et al., 2019). Here, we have isolated this species from *Q. brantii* and *Q. libani* for the first time from Kurdistan, Kermanshah, and Lorestan provinces. *Didymella glomerata* in both pathogenicity experiments was determined as moderately virulent. Recently, pathogenicity of *D. glomerata* on *Q. brantii* seedlings was confirmed (Alidadi et al., 2019). Additionally, *K. variispora* has been isolated from *Q. brantii* as an endophyte in Kohgiluyeh and Boyer-Ahmad (Ghobad-Nejhad et al., 2017) and associated with oak decline in Ilam provinces (Alidadi et al., 2018), while we have isolated this species from all three *Quercus* species (*Q. brantii*, *Q. infectoria*, and *Q. libani*) growing in Zagros forests in Ilam, Kermanshah, Kurdistan, Lorestan, and West Azarbaijan provinces. In pathogenicity experiments, *K. variispora* was moderately and weakly virulent on oak leaves and seedling stems, respectively. Based on our knowledge, the pathogenicity of *K. variispora* is confirmed on oak trees for the first time.

The majority of identified fungi (19 out of 24, 79%), including all coelomycetous species, were determined as pathogenic; thus, it is important to consider them in various aspects of pathogenicity, host range, geographic distribution, genetic diversity, and management. Since phytotoxic secondary metabolites are major biochemical weapons, as pathogenicity or virulence factors in fungal pathogens causing canker, dieback, and decline diseases in woody plants (Evidente et al., 2019), we have studied and characterized phytotoxic compounds of some species such as *B. rosacearum* (Masi et al., 2021), *F. rabenhorstii* (Bashiri et al., 2020a), and *S. banihashemiana*, previously identified as *S. macrostoma* (Di Lecce et al., 2020). At the time being, phytotoxic metabolites of six species, *A. iranensis*, *G. quercicola*, *N. dimidiatum*, *Ph. tuscanicum*, *Cosmospora butyri*, and *Neocosmospora* sp., are being investigated.

In this study, branch canker, dieback, and defoliation were obviously dominant external symptoms of declined oak trees accompanied by borer hole and central and irregular necrosis as common internal symptoms on twigs’ cross-sections. We found no correlation between fungal species isolated and type of internal and external symptoms as we can infer from several studies (Mohammadi et al., 2014; Hashemi and Mohammadi, 2016; Esparham et al., 2020; Soltaninejad et al., 2017; Bashiri et al., 2022). Most of the identified species were isolated in association with borer hole (feeding site of insects) on infected trees; thus, it is necessary to consider the role of insects in transmission, penetration, and emergence of opportunistic fungal pathogens, weakness, and decline of oak trees in combination with fungal species. In the last decades, many fungi have been isolated from insects or feeding sites of larvae including *Biscogniauxia rosacearum*, *B. persica*, *B. dryophila*, *Neocosmospora ewallaceae*, *N. ambrosia*, *N. metavorans*, *Fusarium* spp., *Diplodia corticola*, *Dothiorella iberica*, and *Cryphonectria naterciae* (Pazoutova et al., 2010; O’Donnell et al., 2012; Freeman et al., 2013; Bateman et al., 2016; Herr et al., 2016; Bashiri et al., 2022).

According to the composition and diversity of the identified fungal species, the pathogenicity of the majority of species, and the presence of some well-known and new highly virulent species (e.g., *Biscogniauxia rosacearum*, *Botryosphaeria dothidea*, *N. dimidiatum*, and *G. quercicola* sp. nov.) together with their association with insect borer hole, we strongly recommend to investigate in more detail all identified fungi specifically the most virulent ones and new fungal species to discover their interaction with growing oak species, as well as their relationships together and with insects and abiotic stress. Concurrently, regarding the threats facing Zagros oak forests including drought, heat and dust stresses due to climate change, wildfire, plant diseases (charcoal canker), and pests (borer beetles and green oak tortrix), it is crucial to prevent inappropriate human exploitation and forest management through training and improving welfare of local communities and following environmental friendly approaches dealing with plant diseases and pests.

## Data availability statement

The datasets presented in this study can be found in online repositories. The names of the repository/repositories and accession number(s) can be found in the article/**Supplementary Material**.

## Author contributions

SB: Formal analysis, Investigation, Software, Writing – original draft. JA: Conceptualization, Funding acquisition, Methodology, Project administration, Resources, Supervision, Writing – review & editing.
