# Supplementary material for: Taxonomy and pathogenicity of fungi associated with oak decline in northern and central Zagros forests of Iran with emphasis on coelomycetous species
Source: Front Plant Sci. 2024 Apr 18;15:1377441. doi: 10.3389/fpls.2024.1377441 (PMC11067508; doi:10.3389/fpls.2024.1377441)
Supplement: Supplementary file 1 [file DataSheet_1.docx]

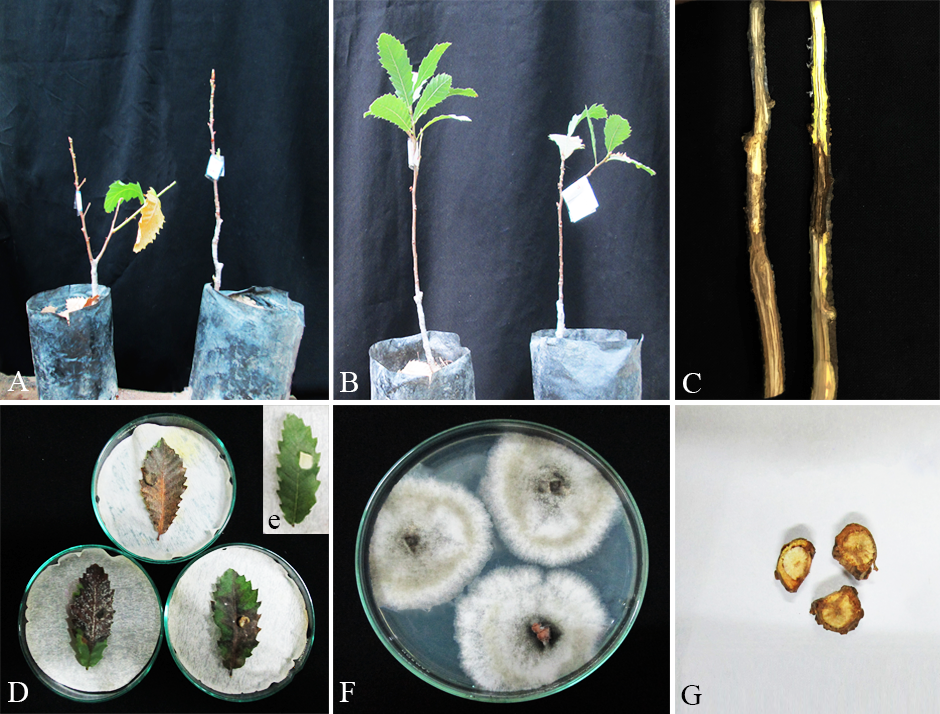


**Supplementary Figure 1.** Pathogenicity tests and disease symptoms caused by *B. dothidea* on oak seedlings in greenhouse **(A–C,G)** and leaves *in vitro* **(D,E)** conditions. **(A)** inoculated plants after one month. **(B)** control. (**C**) necrotic lesion on stems. **(D,E)** necrotic spot on leaves and control. **(F)** *B. dothidea* colony re-isolated from inoculated seedlings. **(G)** stem cross-sections showing wood necrosis and discoloration.


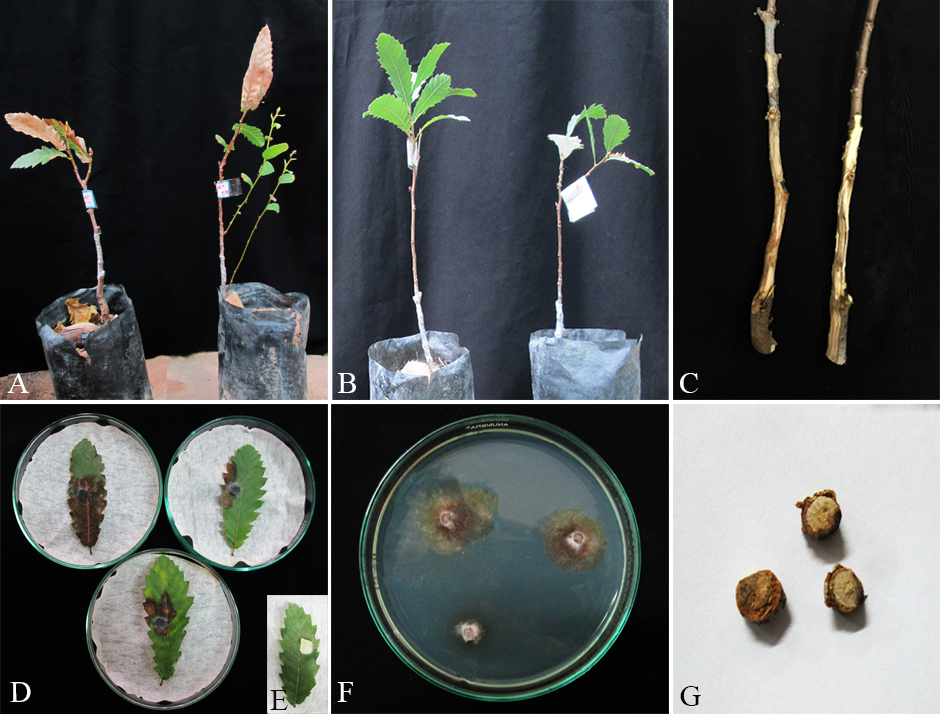


**Supplementary Figure 2.** Pathogenicity tests and disease symptoms caused by *D. glomerata* on oak seedlings in greenhouse **(A–C,G)** and leaves *in vitro* **(D,E)** conditions. **(A)** inoculated plants after 2 months. **(B)** control. (**C**) necrotic lesion on stems. **(D,E)** necrotic spot on leaves and control. **(F)** *D. glomerata* colony re-isolated from inoculated seedlings. **(G)** stem cross-sections showing wood necrosis and discoloration


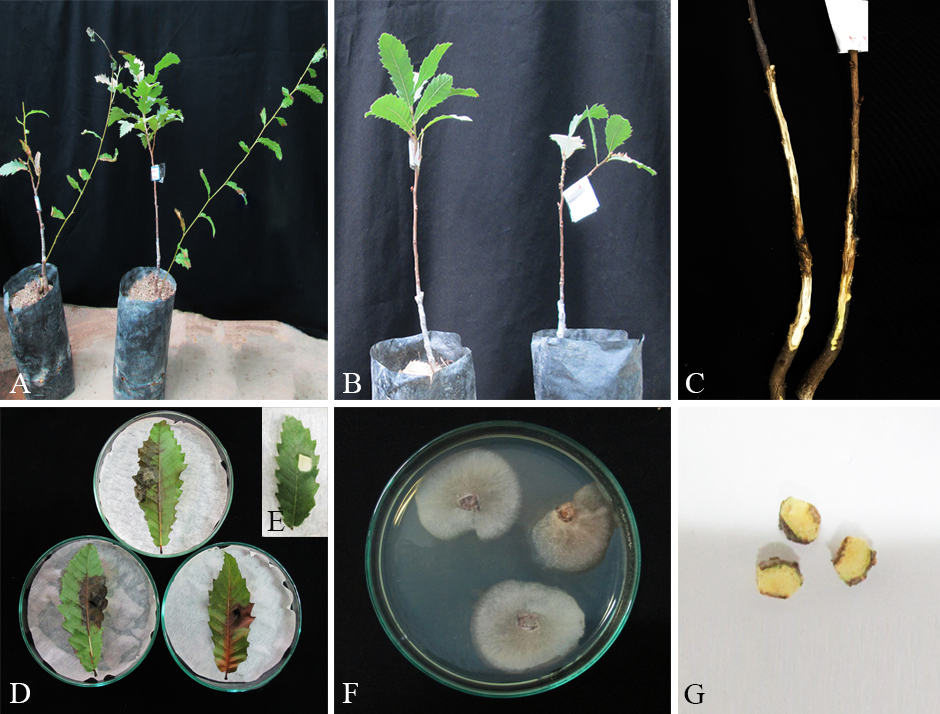


**Supplementary Figure 3.** Pathogenicity tests and disease symptoms caused by *K. variispora* on oak seedlings in greenhouse **(A–C,G)** and leaves *in vitro* **(D,E)** conditions. **(A)** inoculated plants after 2 months. **(B)** control. (**C**) necrotic lesion on stems. **(D,E)** necrotic spot on leaves and control. **(F)** *K. variispora* colony re-isolated from inoculated seedlings. **(G)** stem cross-sections showing wood necrosis and discoloration;


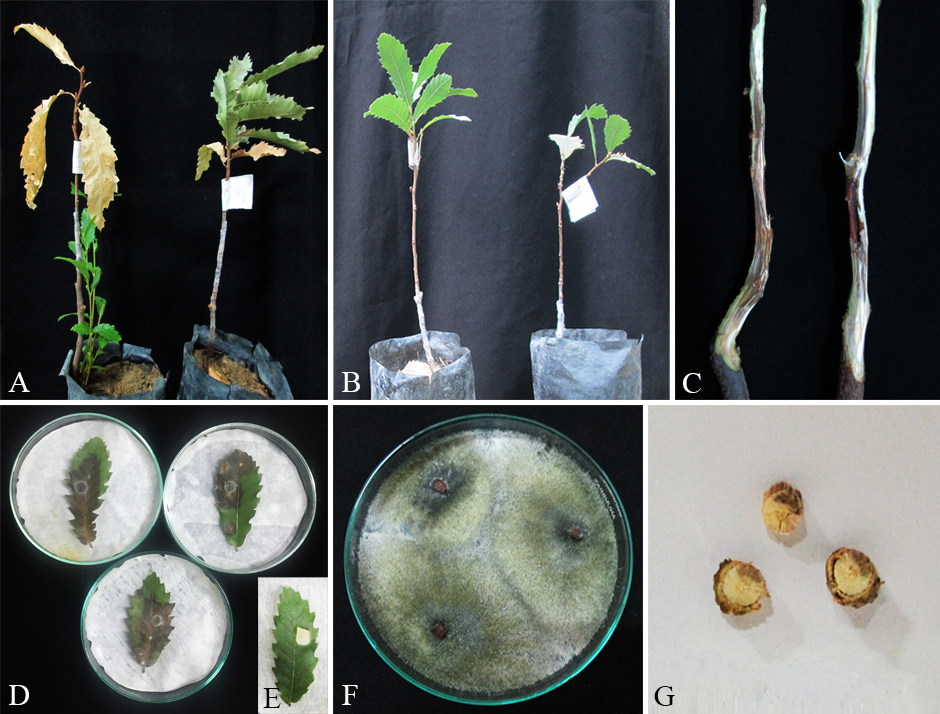


**Supplementary Figure 4.** Pathogenicity tests and disease symptoms caused by *N. dimidiatum* on oak seedlings in greenhouse **(A–C,G)** and leaves *in vitro* **(D,E)** conditions. **(A)** inoculated plants after one month. **(B)** control. (**C**) necrotic lesion on stems. **(D,E)** necrotic spot on leaves and control. **(F)** *N. dimidiatum* colony re-isolated from inoculated seedlings. **(G)** stem cross-sections showing wood necrosis and discoloration.
